# Human sperm acrosome function assays are predictive of fertilization rate in vitro: a retrospective cohort study and meta-analysis

**DOI:** 10.1186/s12958-018-0398-y

**Published:** 2018-08-24

**Authors:** Fang Xu, Ganggang Guo, Wenbing Zhu, Liqing Fan

**Affiliations:** 1 Institute of Reproductive and Stem Cell Engineering, School of Basic Medicine Science, Central South University, Changsha, 410078 China; 2 Business School, Central South University, Changsha, 410078 China; 3 Reproductive and Genetic Hospital of CITIC-Xiangya, Changsha, 410078 China

**Keywords:** Acrosomal function, Acrosomal enzyme, Acrosin, IVF, Retrospective study, Meta-analysis

## Abstract

**Objective:**

To determine whether acrosome function scoring—including acrosomal enzyme (AE) levels and acrosome reaction (AR) results—can predict fertilization rate in vitro.

**Methods:**

We examined the predictive value of acrosomal enzymes (AE) determined by spectrophotometry/*N*-α-benzoyl-dl-arginine-p-nitroanilide for fertilization rate (FR) in vitro in a retrospective cohort study of 737 infertile couples undergoing IVF therapy. Additionally, a meta-analysis was done for prospective cohort or case-control studies; the following summary measures were reported to expand upon the findings: pooled spearman correlation coefficient (Rs), standardized mean difference (SMD), sensitivity (SEN), specificity (SPE), positive likelihood ratio (PLR), negative likelihood ratio (NLR), diagnostic score (DS), diagnostic odds ratio (DOR), and area under the summary receiver operating characteristic curve (AUC).

**Results:**

Lower AE levels determined by spectrophotometry with a cut-off value of <25μIU/10^6^ spermatozoa were predictive of total fertilization failure (TFF) with moderate SEN (88.23%) and low SPE (16.50%). On meta-analysis, a total of 44 unique articles were selected, but given the multiple techniques described there was a total of 67 total datasets extracted from these 44 articles, comprising 5356 infertile couples undergoing IVF therapy. The AE levels or induced AR% was positively correlated with FR (Rs = 0.38, SMD = 0.79; Rs = 0.40, SMD = 0.86, respectively). Lower AE levels or induced AR% was predictive of lower fertilization rate with moderate accuracy (AUC = 0.78, AUC = 0.84, respectively); this was accompanied by low SEN/moderate SPE (0.57/0.85), moderate SEN/moderate SPE (0.79/0.87), respectively. For AE assay, the diagnostic performance in Asia (Rs = 0.24, SMD = 0.50) was inferior to that in North America (Rs = 0.54, SMD = 0.81) and Europe (Rs = 0.46, SMD = 0.92). Cryopreserved spermatozoa (SMD = 0.20, *P* = 0.204) were inferior to fresh spermatozoa (SMD = 0.89, *P* <  0.001). Sperm preparation yielded inferior results as compared to no preparation; spermatozoa after swim up were weak relevant (Rs = 0.27, *P* = 0.044); and there was no correlation for spermatozoa after a discontinuous gradient (SMD = 1.07, *P* >  0.05). Lower AE levels determined by fluorometry or substrate assay were used for predicting lower FR with low sensitivity and high specificity; the spectrophotometry assay had an uncertain predictive value. For induced AR assay, the diagnostic performance in the other areas was inferior to that in Africa (Rs = 0.65, SMD = 1.86). No preparation or double preparation yielded inferior results as compared to one preparation (Rs = 0.41); discontinuous gradient (Rs = 0.17, SMD = 0.47) was inferior to swim up (Rs =0.65, SMD = 1.51). Nonphysiological triggers (SMD = 0.81) did not differ from physiological triggers (SMD = 0.95) in general; ZP (Rs = 0.63) or mannose (Rs = 0.59) was superior to other physiological or nonphysiological triggers; and there was no correlation for human follicle fluid, progesterone, cyclic adenosine 3′-5′-phosphate analogue and phorbol ester–BSA-GlcNAc Neoglycoproteins with N-acetylglucosamine residues. Lower induced AR% determined by indirect immunofluorescence, direct immunofluorescence with lection, or triple stain was used for predicting lower FR, with moderate sensitivity/high specificity, moderate sensitivity/high specificity, or high sensitivity/low specificity.

**Conclusions:**

Although the correlation between acrosome function scoring and FR was significant, the assays were neither highly sensitive nor specific. Additionally, the diagnostic performance showed regional effects as well as an effect of the sperm preparation or assay method. More studies of multicenter, large-scale, careful design and synthesizing multiple sperm functional assays and oocyte quality assays are still needed in clinical settings to better predict fertilization outcome in IVF.

**Electronic supplementary material:**

The online version of this article (10.1186/s12958-018-0398-y) contains supplementary material, which is available to authorized users.

## Background

The sperm acrosome is a Golgi complex-derived flat granule overlaying the anterior two-thirds of the sperm head and contains numerous acrosomal enzymes (AEs) such as protease, glycosidase, acrosin, hyaluronidase, and high-electron density semisolid matrix proteins. Among AEs, the serine proteinase acrosin and hyaluronidase are of particular interest owing to their roles in fertilization, which include limited proteolysis of zona proteins to facilitate spermatozoa penetration into the various layers of the ovum. Acrosin—which is exclusive to the acrosome of mammalian spermatozoa—is mainly synthesized and stored in an enzymatically inactive zymogen form (i.e., proacrosin), and is released during acrosomal exocytosis following maturation [1]. Hyaluronidase is secreted and depolymerizes the matrix between cells of the cumulus oophorus [2].

Intact acrosome function—containing adequate active AEs (proacrosin, acrosin, and hyaluronidase) and ability to undergo acrosome reaction (AR) after the induction—is necessary for sperm fertility. The detection of acrosome function can provide insight into the fertilizing capacity of spermatozoa, and is therefore considered a useful diagnostic tool for male infertility. Several methods have been described to assay AE, including fluorometry, western blotting, spectrophotometry, substrate assays, and radioimmunoassay (RIA). For the indirect fluorometry, polyclonal anti-acrosin (pAb-acrosin) [3] or anti-hyaluronidase (pAb-hyaluronidase) antibodies [3] or a monoclonal anti-proacrosin antibody (mAb 4D4-proacrosin) [4] is used. In addition, anti-acrosin antibody with low binding specificity has been used for western blotting [5]. There are several types of spectrophotometry assay, including an acrosin/proacrosin target with *N*-α-benzoyl-dl-arginine-p-nitroanilide (BAPNA) substrate (spectrophotometry/BAPNA) [6–8]; acrosin/proacrosin target with BAPNA substrate in a commercially available acrosin activity assay kit (Accu-Sperm) (Accu-Sperm spectrophotometry/BAPNA) [9]; acrosin/proacrosin target with N-benzoyl-l-arginine ethyl ester (BAEE) substrate (spectrophotometry/BAEE) [10, 11]; acrosin/proacrosin/acrosin inhibitor target with BAEE substrate [12]; and hyaluronidase target with BAEE substrate [13]. Substrate assays include a hyaluronidase target with cytochemical substrate [14]; acrosin target with gelatine substrate [15–18]; hyaluronidase target with agar/hyaluronic acid mixture substrate [19]; and hyaluronidase target with hyaluronic acid substrate [2]. Finally, an RIA has been used to quantify acrosin in sperm acid extracts irrespective of the presence of acrosin inhibitors [20].

For assessing human sperm AR, three kinds of methods are used, including transmission electron microscopy (TEM), dyes for bright-field microscopy (DBM), fluorescent labels [21]. For the TEM, it is usually the god standard against which a new assay is measured and it cannot be routinely used owing to labor consuming and lack of sperm viability assay [21]. For the DBM, two stain (an acrosomal stain, a nuclear stain) [22] and triple stain (Bismark brown, rose Bengal, trypan blue) [23, 24] are the most widely used. There are three classes of fluorescent labels: those that label permeabilized spermatozoa with internally directed probes, including fluorescein isothiocyanate-conjugated Pisurn sativum agglutimm (FITC-PSA) [25–36], peanut agglutinin (FITC-PNA) [37–39], Concanavalin A lectin (FITC-Con A) [40], GB24 antibody (FITC-GB24) [41, 42], rhodamine-conjugated PSA (RITC-PSA) [43], and tetramethylrhodamine-conjugated PSA (TRITC-PSA) [44]; those that label permeabilized spermatozoa with by indirect immunofluorescence with antibodies—including HS21 [45], HS63 [46], GB24 [37, 47], MH61 [29], anti-CD46 [48]—directed against acrosome-associated antigens; and those—such as chlortetracycline (CTC) [49]—that can be used on living, nonpermeabilized cells.

Conflicting results have been reported concerning the utility of acrosome function scoring determined by different methods for predicting fertilization rate (FR) in vitro. Some studies showed that there was no correlation between acrosome function scoring and FR [9, 10, 41, 50, 51]. In contrast, others have reported a positive correlation between the two parameters by fluorometry [3, 4], spectrophotometry [3, 6–8, 52–58], and substrate assay [2, 15–19]. To clarify this contradiction, we retrospectively investigated the correlation between AE levels determined by spectrophotometry/BAPNA with FR. Additionally, a systematic review and meta-analysis of published literature on similar topic, without regard to acrosome function assay methods, was performed to further expand upon the findings.

## Methods

### Retrospective cohort study

#### Patients

From July 2015 to March 2016, 737 infertile couples undergoing in vitro fertilization (IVF) therapy for whom ≥4 MII oocytes used for fertilization in vitro on the day of therapy, while excluding those presenting for IVF with intracytoplasmic sperm injection (ICSI) therapy, were included in retrospective analysis. The aetiologies of infertility were as follows: male factor in 133 (single problem = 93; oligozoospermia: 6, asthenozoospermia: 38, teratozoospermia: 49; ≥ 2 male problems mentioned above = 40); female factor in 353 (single problem = 195; tubal occlusion: 190, ovulatory disorder: 0, endometriosis: 1, polycystic ovarian syndrome: 0, intrauterine adhesion: 1, uterine myomas: 1, uterine malformation: 0, genital tract malformation: 0, pelvic inflammatory disease: 2, immune infertility: 0, adiposis: 0, hyperlipemia: 0, hyperprolactinemia: 1; ≥ 2 female problems mentioned above = 158); couple factors in 251(≥ 1 male problem and ≥ 1 female problem mentioned above).

#### AE determination

Prior to further inclusion of couples in therapy protocol, the semen samples were collected and AE levels were determined by the procedure of Kennedy [6], with proper modifications. Briefly, the experimental and control tubes, each containing 7.5 ×  10^6^ spermatozoa, were layered over 500 μL of 11% Ficoll (Sigma-Aldrich, St. Louis, MO, USA) and centrifuged at 2000×g for 20 min. Then 100 μL of benzamidine (500 mM, Sigma-Aldrich, St. Louis, MO, USA) was added to equal volume of sperm pellet in the control tube. Afterwards, 1 mL of substrate-detergent mixture (BAPNA-Triton X-100 mixture, PH = 8.0, Sigma-Aldrich, St. Louis, MO, USA) was added to both tubes. After 1 h of incubation at 24 °C, benzamidine (100 μL) was added to experimental tube to stop the reaction. All samples were centrifuged at 2000×*g* for 15 min and the absorbance of supernatants was spectrophotometrically determined at 410 nm. AE activity (μIU/10^6^) was calculated out of the difference in optical density between experimental and control tube of each sample.

### Meta-analysis

#### Data sources and study selection

Two investigators independently carried out a search in PubMed, Web of Science, Cochrane Library, Embase, EBSCO, Ovid, ClinicalTrials.gov and Google Scholar databases for relevant literature up to February 2017. The [Title/Abstract] search was restricted to English language publications and was performed for the following MeSH terms: fertilization in vitro, acrosin, acrosome reaction, exocytosis, predictive value of tests, sensitivity and specificity (Additional file 1: search strategy). Inclusion criteria were as follows: (1) prospective cohort or case-control design; (2) infertile couples undergoing IVF therapy; (3) a study population of at least 30 couples; (4) AE or AR assay as an index test; (5) oocytes examined to establish fertilization as a reference standard test.

#### Data extraction and quality assessment

Information on study characteristics was independently abstracted by two investigators according to a standardized table (Table 2–4), with decisions made by consensus in cases of disagreement. In four articles where there were ≥ 1 outcome indicators, data with a maximal correlation coefficient and corresponding 95% confidence interval (CI) were used [16, 18, 25, 44]. In four articles where there were ≥ 1 AE/AR cut-off values, data with the best sensitivity (SEN) or specificity (SPE) were used [3, 6, 39, 59]. The methodological quality of eligible articles was assessed with the QUADAS-2 tool [60]. Based on user guidelines, items were tailored by omitting or modifying some signaling questions [60]; for example, when reviewing Patient Selection, the item “Was a case–control design avoided?” was omitted; and for a review of Objective Index Test, the item “If a threshold was used, was it pre-specified?” was substituted with “Was the method of determining AEs or AR described?” This substitution was made because candidate articles were included regardless of the method of acrosome function detection.

### Statistical analysis

In retrospective cohort study, the statistical analysis was performed by SPSS version 16.0 for Windows (SPSS Inc., Chicago, IL, USA). Data were presented as number and percentages for categorical variables, while non-normal variables were reported as median and interquartile ranges. Spearman rank analysis was performed to determine which variables were related to FR. The Pearson χ2-test was performed for comparison for the frequencies of categorical variables. Two-tailed *p* <  0.05 was considered statistically significant. In meta-analysis, data analysis was performed using STATA 12.0 software (Stata Corp., College Station, TX, USA). Statistical heterogeneity was evaluated using the Q test or inconsistency index (I^2^), with significance set at *p* <  0.05 or I^2^ >  50%, respectively. If heterogeneity existed, the random effects model was adopted; otherwise, a fixed-effects model was selected. SEN and subgroup analyses were carried out to identify suspected sources of heterogeneity. Subgroups were compared with the Q test for heterogeneity [61]. The bivariate mixed effects regression model of midas module in STATA 12.0 was used for calculating SEN, SPE, positive likelihood ratio (PLR), negative likelihood ratio (NLR), diagnostic score (DS), diagnostic odds ratio (DOR), and for performing the summary receiver operating characteristic (SROC) curve analysis and drawing Fagan nomogram.

## Results

### Retrospective cohort study

The baseline characteristics, AE result, and fertilization rate for the couples included in the analysis are described in Table 1. The sample size retrieved (*n* = 737) for this retrospective study was greater than the calculated values (334–687) for cohort study by Epi Info version 7.2 for Windows (https://www.cdc.gov/epiinfo/pc.html), with two-sided confidence level set at 95%, power set at 90%, ratio (unexposed: exposed) set at 0.1945 (120/617), and the % outcomes in unexposed group set at 5–10% (i.e., the occurrence of total fertilization failure [TFF, FR = 0%] described previously [62]. The median and interquartile range obtained for AE levels was 13.78 μIU/10^6^ spermatozoa (12.12 μIU/10^6^ spermatozoa). The FR was shown to be positively correlated with forward progression motility (spearman *r* = 0.119, *p* = 0.001) and AE levels (spearman *r* = 0.075, *p* = 0.042; Additional file 2: Table S1). According to a previously published report [6], patients were separated into two groups (< 25 μIU/10^6^ spermatozoa, ≥ 25 μIU/10^6^ spermatozoa), based on the AE levels results. Significantly higher FR were obtained in the group with AE activity ≥25 μIU/10^6^ spermatozoa, compared with those with AE activity < 25 μIU/10^6^ spermatozoa (78.98% [1101/1394], *n* = 120 vs. 73.31% [4843/6606], *n* = 617, *p* <  0.001). The lower AE result with a cut-off value of <25μIU/10^6^ spermatozoa was not a risk factor for patients suffering from TFF (risk ratio [RR] = 1.46, 95% CI: 0.52–4.07), and was used for predicting TFF, showing moderate SEN (88.23% [30/34]) and low SPE (16.50% [116/703], Additional file 3: Table S2).

**Table 1:** Baseline characteristics, AE result, and fertilization rate for the couples included in the analysis

Variables	Median (interquartile range)	N
Female age (years)	29 (5)	737
Male age (years)	34 (7)	737
MII oocytes (n)	9 (8)	737
Abstinence days (n)	4 (2)	737
Semen volume (mL)	3.20 (1.5)	737
Concentration (× 10^6^/mL)	42.00 (36)	737
Motility (%)	50.00 (12.6)	737
Forward progression motility (%)	35.10 (9.1)	737
Percentage of normal morphology (%)	4.46 (0.94)	737
Infertility duration (years)	4 (2)	737
Infertile diagnoses, n (%)		
Male factor	133 (18.05%)	737
Female factor	353 (47.90%)	
Couple factors	251 (34.05%)	
Infertile types, n (%)		
Primary infertility	476 (64.59%)	737
Secondary infertility	261 (35.41%)	
Ovulation inducing protocols, n (%)		
Conventional long pituitary downregulation protocol	35 (4.75%)	737
Modified ultra-long pituitary downregulation protocol	702 (95.25%)	
AE levels (μIU/10^6^ spermatozoa)	13.78 (12.12)	737
FR^a^ (%)	74.30 (5944/8000)	737

*AE* acrosomal enzyme, *MII* metaphase II, *IVF* in vitro fertilization, *FR* fertilization rate

^a^Total fertilized oocytes/total MII oocytes

### Meta-analysis

#### Literature search results

**Fig. 1:**
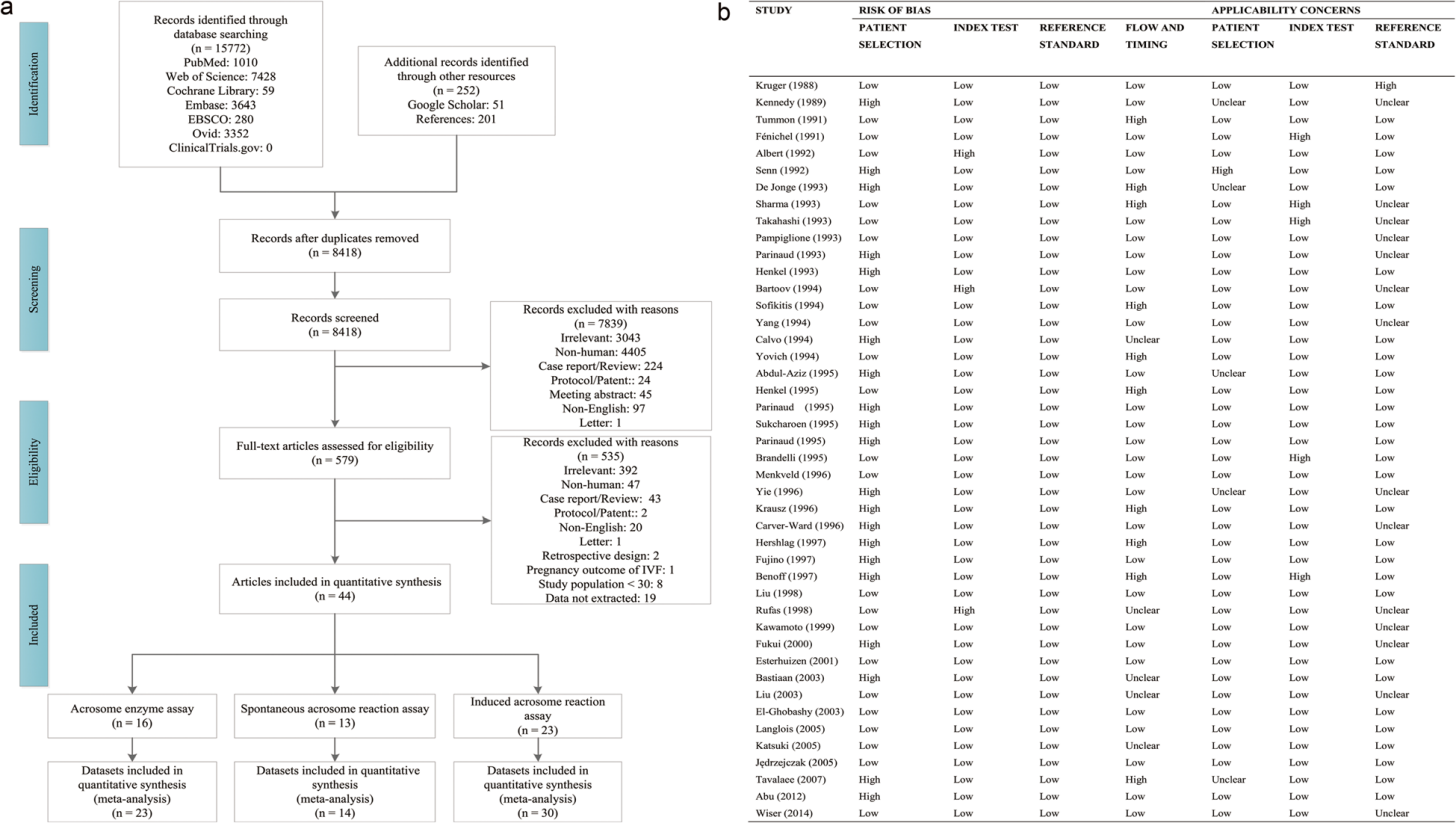
**a, b** Flowchart of the study selection process (**a**). Methodological quality of eligible articles evaluated with the QUADAS 2 tool (**b**). All included articles comprised at least four items of low bias, indicating high overall quality

We initially identified 16,024 candidate articles through database searches (*n* = 15,772) and additional records (*n* = 252). After removing 7606 duplicates, we browsed the titles and abstracts of 8418 articles and selected 579 for full-text reading. The reasons for excluding the others were as follows: irrelevant (*n* = 3043); non-human (*n* = 4405); case report/review (*n* = 224); protocol/patent: (*n* = 24; protocol: 21, patent: 3); meeting abstract (*n* = 45); and non-English (*n* = 97; Chinese: 75, Iranian: 1, French: 3, Japanese: 16, German: 2), and Letter (n = 1). Of the 44 selected articles, 16 articles [3, 4, 6–9, 16, 18, 19, 50, 51, 53–57] addressed the relationship between the AE levels and FR (Table 2); one described three AE assay methods [3]; another reported three sperm preparation methods [54]; and three also mentioned different preparation methods [4, 7, 9] for a total of 23 total datasets extracted from these 16 articles, comprising 2734 infertile couples undergoing IVF therapy. A total of 13 articles [22, 29, 33, 36, 37, 39–42, 44, 47, 63, 64] addressed the relationship between the spontaneous AR% and FR (Table 3); one described two AR assay methods [37] for a total of 14 total datasets extracted from these articles, comprising 791 infertile couples. A total of 23 articles [23–28, 30–35, 37–39, 41–43, 47, 48, 59, 63, 64] addressed the relationship between the induced AR% and FR (Table 4); one described two AR assay methods [37]; another reported five AR triggers [41]; and two also mentioned different triggers [39, 42] for a total of 30 total datasets extracted from these articles, comprising 1831 infertile couples (Fig. 1a).

**Table 2:** Characteristics of datasets that addressed the relationship between the AE levels and FR

First author and year	Country	Design	N	Preparation method	Storage method	AE assay method	FR cut-off value	AE cut-off value	Outcome
Kruger 1988 [50]	USA	Prospective cohort	60	No preparation	Cryopreservation	Spectrophotometry/BAEE	> 0% vs. = 0%^②^		②
Kennedy 1989 [6]	USA	Prospective cohort	35	No preparation	Fresh	Spectrophotometry/BAPNA	> 0% vs. = 0%^②^ /= 0%^③^	< 25μIU/10^6^ spermatozoa^③^	②/③
Tummon 1991a [7]	USA	Prospective cohort	87	No preparation	Fresh	Accu-Sperm Spectrophotometry/BAPNA	> 0% vs. = 0%^②^ /= 0%^③^	< 4.5^③^	②/③
Tummon 1991b [7]	USA	Prospective cohort	87	Swim up	Fresh	Accu-Sperm Spectrophotometry/BAPNA	> 0% vs. = 0%^②^		②
Albert 1992a [4]	France	Prospective case-control	50	No preparation	Fresh	MAb 4D4-proacrosin	Whole^a①^ /> 0% vs. = 0%^②^ /= 0%^③^	≤ 50%^③^	①/②/③
Albert 1992b [4]	France	Prospective case-control	50	Swim up	Fresh	MAb 4D4-proacrosin	> 0% vs. = 0%^②^		②
Senn 1992a [3]	Switzerland	Prospective cohort	151	Discontinuous gradient	Fresh	PAb-acrosin	> 0% vs. =0%^②^ /= 0%^③^	< 60%^③^	②/③
Senn 1992b [3]	Switzerland	Prospective cohort	148	Discontinuous gradient	Fresh	PAb-hyaluronidase	> 0% vs. = 0%^②^ /= 0%^③^	< 80%^③^	②/③
Senn 1992c [3]	Switzerland	Prospective cohort	96	Discontinuous gradient	Fresh	Spectrophotometry/BAPNA	= 0%^③^	< 30 μIU/10^6^ spermatozoa^③^	③
De Jonge 1993 [8]	USA	Prospective cohort	21^①^/60^②^	No preparation	Fresh	Spectrophotometry/BAPNA	> 70%^b①^ /≥ 70% vs. < 70%^②^	< 12.8 μIU/10^6^ spermatozoa	①/②
Sharma 1993a [54]	UK	Prospective cohort	46	Swim up	Fresh	Spectrophotometry/BAPNA	Whole		①
Sharma 1993b [54]	UK	Prospective cohort	31	Discontinuous gradient	Fresh	Spectrophotometry/BAPNA	Whole		①
Sharma 1993c [54]	UK	Prospective cohort	25	α-Chymotrypsin	Fresh	Spectrophotometry /BAPNA	Whole		①
Bartoov 1994 [57]	Israel	Prospective case-control	59	No preparation	Fresh	Spectrophotometry /BAPNA	Whole^①^ /> 50% vs. = 0%^②^/= 0%^③^	≤ 54 μIU/10^6^ spermatozoa^③^	①/②/③
Sofikitis 1994 [51]	USA	Prospective cohort	41	No preparation	Fresh	Accu-Sperm Spectrophotometry /BAPNA	> 0% vs. = 0%^②^		①
Yang 1994a [9]	China	Prospective cohort	95	No preparation	Fresh	Accu-Sperm Spectrophotometry /BAPNA	Whole^①^ /> 0% vs. = 0%^②^		①/②
Yang 1994b [9]	China	Prospective cohort	95	Swim up	Fresh	Accu-Sperm Spectrophotometry /BAPNA	Whole^①^ /> 0% vs. = 0%^②^		①/②
Abdul-Aziz 1995 [19]	Canada	Prospective cohort	160	No preparation	Fresh	Hyaluronidase target with agar/hyaluronic acid mixture substrate	Whole^①^	≤ 3 mm	①
Henkel 1995 [16]	Germany	Prospective cohort	110	Swim up	Fresh	Acrosin target with gelation substrate assay	whole^①^/ ≤ 50%^③^	< 6^③^	①/③
Menkveld 1996 [55]	Netherlands	Prospective cohort	33	No preparation	Fresh	Spectrophotometry /BAPNA	whole^①^ /≥ 50% vs. < 50%^②^/< 50%^③^	≤ 18 μIU/10^6^ spermatozoa^*③*^	①/②/③
Yie 1996 [53]	Canada	Prospective cohort	259	Swim up	Cryopreservation	Spectrophotometry /BAPNA	> 0% vs. 0%^②^ /= 0%^③^	≤ 15 μIU/ μgDNA^③^	②/③
Langlois 2005 [56]	Belgium	Prospective cohort	107	No preparation	Fresh	Spectrophotometry /BAPNA	whole^①^ /≥ 50% vs. < 50%^②^/< 50%^③^	< 25 μIU/10^6^ spermatozoa^*③*^	①/②/③
Tavalaee 2007 [18]	Iran	Prospective cohort	48	Discontinuous gradient	Fresh	Acrosin target with gelation substrate assay	whole^①^ /< 50%^③^	< 60%^③^	①/③

*AE* acrosomal enzyme, *FR* fertilization rate, *BAEE N*-benzoyl-l-arginine ethyl ester, *BAPNA N*-α-benzoyl-dl-arginine-p-nitroanilide

^a^All infertile patients undergoing in vitro fertilization (IVF) therapy

^b^Infertile patients for whom ≥70% fertilization was achieved by IVF

①spearman correlation coefficient; ② AE levels for higher and lower FR; ③ binary diagnostic accuracy data as 2 × 2 tables

**Table 3:** Characteristics of datasets that addressed the relationship between the spontaneous AR% and FR

First author and year	Country	Design	N	Preparation method	Assay method	FR cut-off value	AR cut-off value	Outcome
Fénichel 1991 [47]	France	Prospective cohort	41	Swim up	GB24	> 0% vs. = 0%		②
Takahashi 1993 [40]	Japan	Prospective cohort	45	Discontinuous gradient	FITC-Con A	< 50%^③^	%AR ≥ 10% at 2 h or ≤ 5% at 4 h^③^	③
Parinaud 1993a [37]	France	Prospective cohort	53	Discontinuous gradient	FITC-PNA	> 0% vs. = 0%^②^		②
Parinaud 1993b [37]	France	Prospective cohort	53	Discontinuous gradient	GB24	> 0% vs. = 0%^②^		②
Parinaud 1995 [41]	Monaco	Prospective cohort	117	Discontinuous gradient	F1TC-GB24	Whole^a^		①
Parinaud 1995 [42]	Monaco	Prospective cohort	131	Discontinuous gradient	FITC-GB24	> 0% vs. = 0%^②^		②
Krausz 1996 [39]	Italy	Prospective cohort	59	Swim up	FITC-PNA	≥ 50% vs. < 50%^②^		②
Hershlag 1997 [44]	USA	Prospective cohort	74	Swim up	TRITC-PSA	Whole^①^		①
Fujino 1997 [22]	Japan	Prospective cohort	30	Discontinuous gradient + Swim up	Two stain (new-methylene blue and cresyl violet) Blutstan kit	Whole^①^		①
Rufas 1998 [64]	Israel	Prospective cohort	62	Discontinuous gradient	FITC-PSA	> 0% vs. = 0%^②^		②
Kawamoto 1999 [29]	Japan	Prospective cohort	34	Swim up	MH61	> 0% vs. = 0%^②^		②
Bastiaan 2003 [63]	South Africa	Prospective cohort	30	Swim up	FITC-PSA	> 50% vs. < 50%^②^		②
El-Ghobashy 2003 [33]	UK	Prospective cohort	75	Swim up	FITC-PSA	≥ 50% vs. < 50%^②^		②
Wiser 2014 [36]	Israel	Prospective cohort	40	Not reported	FITC-PSA	≤ 35%^③^	≤10%^③^	③

*AR* acrosome reaction, *FR* fertilization rate, *FITC-ConA* fluorescein isothiocyanate*-*conjugated Concanavalin A lectin, *FITC-PNA* fluorescein isothiocyanate*-*conjugated peanut agglutinin, *FITC-GB24* fluorescein isothiocyanate*-*GB24, *TRITC-PSA* tetramethylrhodamine-conjugated Pisurn sativum agglutimm, *FITC-PSA* fluorescein isothiocyanate-conjugated Pisurn sativum agglutimm

^a^All infertile patients undergoing in vitro fertilization (IVF) therapy

① spearman correlation coefficient; ② spontaneous AR% for higher and lower FR; ③ binary diagnostic accuracy data as 2 × 2 tables

**Table 4:** Characteristics of datasets that addressed the relationship between the induced AR% and FR

First author and year	Country	Design	N	Preparation method	Inducer	Assay method	FR cut-off value	AR cut-off value	Outcome
Fénichel 1991 [47]	France	Prospective cohort	41	Swim up	A23187	GB24	> 0% vs. = 0%^②^ /= 0%^③^	Spontaneous AR% ≥ 11.6% and/or Induced AR% < 20.6%^③^	②/③
Pampiglione 1993 [23]	UK	Prospective cohort	54	Swim up	A23187	Triple stain	> 0% vs. = 0%^②^ /= 0%^③^	< 31.3%^③^	②/③
Parinaud 1993a [37]	France	Prospective cohort	53	Discontinuous gradient	A23187	FITC-PNA	> 0% vs. = 0%^②^		②
Parinaud 1993b [37]	France	Prospective cohort	53	Discontinuous gradient	A23187	GB24	> 0% vs. = 0%^②^		②
Henkel 1993 [59]	Germany	Prospective cohort	74	Swim up	Low temperature	Triple stain	< 50%^③^	≤ 10%^③^	③
Calvo 1994 [25]	USA	Prospective cohort	82^①^ /232^③^	No preparation	HFF	FITC-PSA	Whole^a①^/= 0%^③^	≤ 5%^③^	①/③
Yovich 1994 [26]	Australia	Prospective cohort	52	Swim up /Discontinuous gradient	A23187	FITC-PSA	= 0%^③^	< 10%^③^	③
Parinaud 1995a [41]	Monaco	Prospective cohort	117	Discontinuous gradient	A23187	FITC-GB24	Whole^①^ /> 0% vs. = 0%^②^		①/②
Parinaud 1995b [41]	Monaco	Prospective cohort	117	Discontinuous gradient	P	FITC-GB24	Whole^①^ /> 0% vs. = 0%^②^		①/②
Parinaud 1995c [41]	Monaco	Prospective cohort	117	Discontinuous gradient	HFF	FITC-GB24	Whole^①^ /> 0% vs. = 0%^②^		①/②
Parinaud 1995d [41]	Monaco	Prospective cohort	117	Discontinuous gradient	CAMP	FITC-GB24	Whole^①^ /> 0% vs. = 0%^②^		①/②
Parinaud 1995e [41]	Monaco	Prospective cohort	117	Discontinuous gradient	TPA	FITC-GB24	Whole^①^ /> 0% vs. = 0%^②^		①/②
Sukcharoen 1995 [38]	UK	Prospective cohort	41	Discontinuous gradient	A23187	FITC-PNA	> 0% vs. < 50%^②^		②
Parinaud 1995a [42]	Monaco	Prospective cohort	131	Discontinuous gradient	A23187	FITC-GB24	> 0% vs. = 0%^②^		②
Parinaud 1995b [42]	Monaco	Prospective cohort	131	Discontinuous gradient	TPA	FITC-GB24	> 0% vs. = 0%^②^		②
Brandelli 1995 [27]	Argentina	Prospective cohort	31	Discontinuous gradient	BSA-GlcNAc/A23187	FITC-PSA	Whole^①^ /≥ 30% vs. < 30%^②^ /< 30%^③^	< 0.2^b③^	①/②/③
Krausz 1996a [39]	Italy	Prospective cohort	53^①^/51^②^ /52^③^	Swim up	A23187	FITC-PNA	Whole^①^ /≥ 50% vs. < 50%^②^ /= 0%^③^	≤ 10%^③^	①/②/③
Krausz 1996b [39]	Italy	Prospective cohort	59^①^ /59^②^ /60^③^	Swim up	P	FITC-PNA	Whole^①^ /≥ 50% vs. < 50%^②^ /= 0%^③^	≤ 7%^③^	①/②/③
Carver-Ward 1996 [48]	Netherlands	Prospective cohort	129	Discontinuous gradient	A23187	Anti-CD46 antibody	Whole^①^ /≤ 30%	≤ 10%^③^	①/③
Benoff 1997 [43]	USA	Prospective cohort	58	Swim up	Mannose	RITC-PSA	Whole^①^ /≥ 63% vs. < 63%^②^ /< 63%^③^	≤ 0.1^c③^	①/②/③
Liu 1998 [28]	Australia	Prospective cohort	109	Discontinuous gradient	A23187	FITC-PSA	Whole^①^ /> 50% vs. ≤ 50%^②^		①/②
Rufas 1998 [64]	Israel	Prospective case-control	62	Discontinuous gradient	HFF	FITC-PSA	> 0% vs. = 0%^②^		②
Fukui 2000 [30]	Japan	Prospective cohort	39	Discontinuous gradient	P	FITC-PSA	> 0% vs. = 0%^②^		②
Esterhuizen 2001 [31]	South Africa	Prospective cohort	35	Swim up	ZP	FITC-PSA	Whole^①^ /≤ 60%^③^	≤ 15%^③^	①/③
Bastiaan 2003 [63]	South Africa	Prospective cohort	30	Swim up	ZP	FITC-PSA	> 50% vs. < 50%^②^ /≤ 50%^③^	< 8%^③^	②/③
Liu 2003 [32]	Australia	Prospective cohort	65	Swim up	ZP	FITC-PSA	Whole^①^ /< 30%^③^	≤ 16%^③^	①/③
El-Ghobashy 2003 [33]	UK	Prospective cohort	75	Swim up	HFF	FITC-PSA	Whole^①^ /≥ 50% vs. < 50%^②^		①/②
Katsuki 2005 [34]	Japan	Prospective cohort	133	Swim up	A23187	FITC-PSA	> 0% vs. = 0%^②^ /= 0%^③^	< 21%^③^	②/③
Jędrzejczak 2005 [24]	Poland	Prospective cohort	79	Discontinuous gradient	A23187	Triple stain	Whole^①^		①
Abu 2012 [35]	South Africa	Prospective cohort	78	Double Swim up	ZP	FITC-PSA	Whole^①^		①

*AR* acrosome reaction, *FR* fertilization rate, *HFF* human follicle fluid, *P* progesterone, *ZP* zona pellucida, *CAMP* cyclic adenosine 3′-5′-phosphate analogue, *TPA* phorbol ester, *BSA-GlcNAc* Neoglycoproteins with N-acetylglucosamine residues, *FITC-PNA* fluorescein isothiocyanate*-*conjugated peanut agglutinin, *FITC-PSA* fluorescein isothiocyanate-conjugated Pisurn sativum agglutimm, *FITC-GB24* fluorescein isothiocyanate*-*GB24, *RITC-PSA* rhodamine-conjugated Pisurn sativum agglutimm

^a^All infertile patients undergoing in vitro fertilization (IVF) therapy

^b^BSA-GlcNAc induced increased %AR/A23187 induced increased %AR

^c^Mannose induced increased %AR/Mannose induced total %AR

① spearman correlation coefficient; ② induced AR% for higher and lower FR; ③ binary diagnostic accuracy data as 2 × 2 tables

#### Study characteristics

All included 44 articles comprised at least four items of low bias in QUADAS 2, indicating high overall quality (Fig. 1b). Forty-one had a prospective cohort design and three had a prospective case-control design. Geographic areas included Asia (*n* = 10), North America (n = 10), Europe (*n* = 17), Africa (*n* = 3), Oceania (*n* = 2), and South America (n = 2). Sperm storage methods included fresh samples (*n* = 41, for AE assay: 13, for AR assay: 28) and cryopreservation (n = 3; for AE assay: 3, for AR assay: 0). Sperm preparation methods included no preparation (*n* = 12), one preparation (*n* = 34; α-chymotrypsin: 1; swim up: 18; discontinuous gradient: 14; swim up/discontinuous gradient: 1;), double preparation (*n* = 2; swim up after discontinuous gradient: 1; double swim up: 1), and not reported (n = 1). AE assay methods included fluorometry (n = 3; pAb-acrosin: 1, pAb-hyaluronidase: 1, mAb 4D4-proacrosin: 1), spectrophotometry (*n* = 13; spectrophotometry/BAPNA: 9, Accu-Sperm spectrophotometry/BAPNA: 3, spectrophotometry/BAEE: 1), and substrate assay (n = 3; acrosin target with gelatine substrate assay: 2, hyaluronidase target with agar/hyaluronic acid mixture substrate assay: 1). All spectrophotometry in the 16 articles had acrosin/proacrosin as targets. AR triggers included physiological triggers (*n* = 10; human follicle fluid [HFF]: 4, progesterone [P]: 3, zona pellucida [ZP]: 3,) and nonphysiological triggers (*n* = 18; calcium ionophore A23187: 12, low temperature: 1, cyclic adenosine 3′-5′-phosphate analogue [CAMP]: 1, phorbol ester [TPA]: 2, Neoglycoproteins with N-acetylglucosamine residues [BSA-GlcNAc]: 1, mannose: 1). AR assay methods included DBM (*n* = 4, two stain Blutstan kit: 1, triple stain: 3) and fluorescent labels (*n* = 24; direct immunofluorescence with lectin: FITC-PSA: 12, FITC-PNA: 3, FITC-ConA: 1, RITC-PSA: 1, TRITC-PSA:1; direct immunofluorescence with antibody: FITC-GB24: 3; indirect immunofluorescence: GB24 antibody: 1, anti-CD46 antibody: 1, MH61 antibody: 1).

#### Data synthesis and analysis

Engauge Digitizer software (http://markummitchell.github.io/engauge-digitizer/) was used to convert the scatter plots in seven articles [6, 7, 18, 27, 38, 39, 63] into coordinates to indirectly obtain acrosome function scoring and FRs. Pearson correlation coefficient from thirteen studies [4, 8, 9, 18, 22, 24, 31, 39, 41, 43, 44, 54, 55] was converted into spearman correlation coefficient (Rs) values followed by Fisher’s r-to-z and z-to-r transformation.

#### AE assay

Rs was extracted from 10 articles that included a total of 758 infertile couples. A total of 13 datasets were analyzed, including one article each that used three [54] and two [9] sperm preparation methods. AE levels and FRs that were higher and lower than the respective cut-off values were extracted from 12 articles, which included a total of 1037 infertile couples. Of the 16 datasets analyzed, one used two AE assay methods [3] and three used two sperm preparation methods [4, 7, 9]. Binary accuracy data from 939 infertile couples were extracted from 10 articles as 2 × 2 tables. We analyzed the 12 datasets, including one paper that used three assay methods [3] (Table 2).

**Fig. 2:**
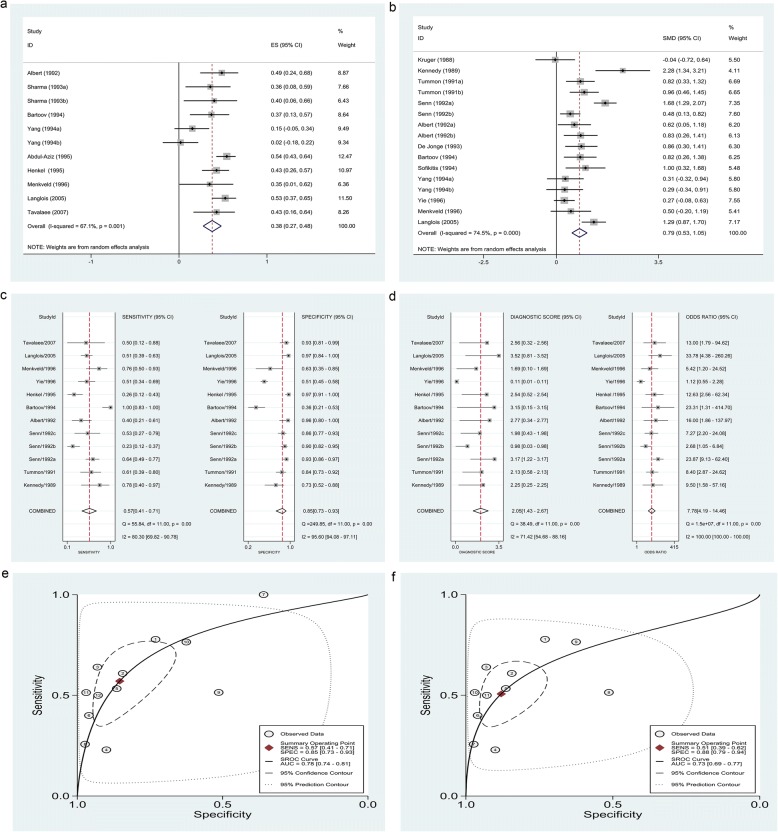
**a–d** Forest plots of Rs (**a**), SMD (**b**), SEN and SPE (**c**), DS and DOR (**d**). (**e, f**) Graphs of SROC curve before (**e**) and after (**f**) excluding one outlier. AE levels were positively correlated with FR (pooled Rs = 0.38). Higher AE levels were obtained for higher as compared to lower FRs (pooled SMD = 0.79). Lower AE levels were predictive of lower FR with low SEN/moderate SPE (pooled SEN = 0.57; SPE = 0.85), moderate discriminant effect (pooled DS = 2.05; DOR = 7.78) and moderate accuracy (AUC = 0.78; AUC = 0.73). *AE* acrosomal enzyme, *Rs* spearman correlation coefficient, *SMD* standardized mean difference, *SEN* sensitivity, SPE specificity, *DS* diagnostic score, *DOR* diagnostic odds ratio, *SROC* summary receiver operating characteristic, *AUC* area under the SROC curve

According to a random-effects model, AE levels were positively correlated with FR (Rs = 0.39, 95% CI: 0.18–0.60, *p* <  0.001), albeit with notable heterogeneity (I^2^ = 95.7%, *p* <  0.001; Table 3; Fig. 2a, Table 5). Higher AE levels were obtained for higher as compared to lower FRs (standardized mean difference [SMD] = 0.79, 95% CI: 0.53–1.05, *p* <  0.001; Fig. 2b, Table 6). The bivariate mixed effects regression model predicted lower FR for lower AE levels with pooled low SEN/moderate SPE (SEN = 0.57, 95% CI: 0.41–0.71; SPE = 0.85, 95% CI: 0.73–0.93), moderate discriminant effect (PLR = 3.91, 95% CI: 2.31–6.61; NLR = 0.50, 95% CI: 0.37–0.68; DS = 2.05, 95% CI: 1.43–2.67; DOR = 7.78, 95% CI: 4.19–14.46) and moderate accuracy (area under the SROC curve [AUC] = 0.78, 95% CI: 0.74–0.81; Fig. 2c–e, Table 7). The Fagan nomogram showed that lower AE levels could be used to predict lower FR when the pre-test probability was 27% (i.e., occurrence rate of patients for whom < 70% fertilization was achieved by IVF in our hospital), with a post-test probability of 59%.

**Table 5:** Summary of Rs values for AE assay

Subgroup	N	R_s_ (95% CI)	Test(s) of heterogeneity		Significance test(s) of R_s_ = 0		*P* ^a^
			I^2^ (%)	*P*	Z	*P*	
Overall	13	0.39 (0.18–0.60)	95.7	0.000	3.70	0.000	
Two experiments excluded	11	0.38 (0.27–0.48)	67.1	0.001	7.19	0.000	
Geographic area							
Asia	4	0.24 (0.05–0.42)	66.7	0.029	2.49	0.013	< 0.001
North America	1	0.54 (0.43–0.65)	–^b^	–	9.70	0.000	
Europe	6	0.46 (0.37–0.54)	0.0	0.798	11.02	0.000	
Preparation method							
No preparation	6	0.42 (0.30–0.55)	63.4	0.018	6.72	0.000	0.04
Swim up	3	0.27 (0.01–0.53)	80.6	0.006	2.01	0.044	
Discontinuous gradient	2	0.42 (0.23–0.60)	0.0	0.893	4.41	0.000	
AE assay method							
Fluorometry							
MAb 4D4-proacrosin	1	0.49 (0.27–0.70)	–	–	4.43	0.000	< 0.001
Spectrophotometry							
Spectrophotometry/BAPNA	5	0.44 (0.35–0.54)	0.0	0.618	9.08	0.000	
Accu-Sperm spectrophotometry/BAPNA	2	0.09 (−0.05–0.23)	0.0	0.352	1.22	0.223	
Substrate assay							
Acrosin target with gelatine substrate assay	2	0.43 (0.30–0.56)	0.0	0.997	6.51	0.000	
Hyaluronidase target with agar/hyaluronic acid mixture substrate assay	1	0.54 (0.43–0.65)	–	–	9.70	0.000	

*Rs* spearman correlation coefficient, *CI* confidence interval, *BAPNA N*-α-benzoyl-dl-arginine-p-nitroanilide

^a^Comparison between subgroups with the Q test for heterogeneity

^b^Not calculated

**Table 6:** Summary of SMD values for AE assay

Subgroup		N	SMD (95% CI)	Test(s) of heterogeneity		Significance test(s) of SMD = 0		*P* ^a^
				I^2^ (%)	*P*	Z	*P*	
Overall		16	0.79 (0.53–1.05)	74.5	0.000	5.97	0.000	
Four experiments excluded		12	0.75 (0.57–0.93)	26.5	0.184	8.19	0.000	
Geographic areas								
Asia		3	0.50 (0.15–0.85)	2.3	0.359	2.77	0.006	0.026
North America		7	0.81 (0.39–1.22)	74.2	0.001	3.80	0.000	
Europe		6	0.92 (0.48–1.36)	80.8	0.000	4.08	0.000	
Storage method								
Cryopreservation		2	0.20 (−0.11–0.52)	0.0	0.425	1.27	0.204	< 0.001
Fresh		14	0.89 (0.63–1.15)	69.3	0.000	6.73	0.000	
Preparation method								
No preparation		10	0.82 (0.50–1.13)	63.1	0.004	5.14	0.000	0.023
Swim up		4	0.57 (0.20–0.94)	53.6	0.091	3.04	0.002	
Discontinuous gradient		2	1.07 (−0.10–2.25)	95.1	0.000	1.79	0.074	
FR cut-off value combined with AE assay method								
≥ 70% vs. < 70%	Spectrophotometry							NC
	Spectrophotometry/BAPNA	1	0.86 (0.30–1.41)	–^b^	–	3.03	0.002	
≥ 50% vs. < 50%	Spectrophotometry							
	Spectrophotometry/BAPNA	2	0.94 (0.18–1.71)	72.5	0.056	2.41	0.016	
> 50% vs. = 0%	Spectrophotometry							
	Spectrophotometry/BAPNA	1	0.82 (0.26–1.38)	–	–	2.87	0.004	
> 0% vs. = 0%	Fluorometry							
	PAb-acrosin	1	1.68 (1.29–2.07)			8.45	0.000	
	PAb-hyaluronidase	1	0.48 (0.13–0.82)			2.68	0.007	
	MAb 4D4-proacrosin	2	0.72 (0.32–1.13)	0.0	0.600	3.50	0.000	
	Spectrophotometry							
	Spectrophotometry/BAPNA	2	1.23 (−0.74–3.19)	93.5	0.000	1.22	0.221	
	Accu-Sperm spectrophotometry/BAPNA	5	0.70 (0.41–0.99)	22.5	0.271	4.70	0.000	
	Spectrophotometry/BAEE	1	−0.04 (−0.72–0.64)	–	–	0.12	0.908	

*SMD* standardized mean difference, *CI* confidence interval, *BAPNA N*-α-benzoyl-dl-arginine-p-nitroanilide, *BAEE N*-benzoyl-l-arginine ethyl ester, *NC* not compared

^a^Comparison between subgroups with the Q test for heterogeneity

^b^Not calculated

**Table 7:** Summary of SEN, SPE, PLR, NLR, DS, DOR, and AUC values for AE assay

Subgroup		N	SEN (95% CI)	SPE (95% CI)	PLR (95% CI)	NLR (95% CI)	DS (95% CI)	DOR (95% CI)	AUC (95% CI)	*P* ^a^
Overall		12	0.57 (0.41–0.71)	0.85 (0.73–0.93)	3.91 (2.31–6.61)	0.50 (0.37–0.68)	2.05 (1.43–2.67)	7.78 (4.19–14.46)	0.78 (0.74–0.81)	
Outlier excluded		11	0.51 (0.39–0.62)	0.88 (0.79–0.94)	4.22 (2.42–7.36)	0.56 (0.45–0.69)	2.02 (1.37–2.67)	7.53 (3.92–14.47)	0.73 (0.69–0.77)	
Storage method										
Cryopreservation		1	0.51	0.51	1.06	0.95	−^c^	1.12	–	< 0.001
Fresh		11	0.60 (0.41–0.76)	0.87 (0.77–0.93)	4.64 (3.04–7.08)	0.46 (0.32–0.68)	2.30 (1.80–2.80)	9.99 (6.05–16.49)	0.83 (0.80–0.86)	
Preparation method										
No preparation		6	0.72 (0.50–0.87)	0.80 (0.59–0.92)	3.68 (1.90–7.13)	0.35 (0.21–0.59)	2.36 (1.71–3.01)	10.56 (5.51–20.26)	0.83 (0.79–0.86)	0.006
Swim up		4^b^	0.38 (0.24–0.54)	0.86 (0.49–0.97)	2.63 (0.75–9.18)	0.73 (0.62–0.85)	1.29 (− 0.06–2.63)	3.62 (0.94–13.90)	0.51 (0.47–0.56)	
Discontinuous gradient		4	0.46 (0.28–0.65)	0.90 (0.87–0.93)	4.83 (2.64–8.83)	0.60 (0.41–0.87)	2.09 (1.14–3.04)	8.08 (3.13–20.88)	0.90 (0.87–0.92)	
FR cut-off value combined with AE assay method										
< 50%	Spectrophotometry									NC
	Spectrophotometry/BAPNA	4^b^	0.63 (0.48–0.76)	0.87 (0.60–0.97)	4.96 (1.51–16.37)	0.42 (0.32–0.57)	2.46 (1.24–3.67)	11.68 (3.47–39.36)	0.75 (0.71–0.79)	
	Substrate assay									
	Acrosin target with gelatine substrate assay	1	0.50	0.93	7.00	0.54	–	13.00	0.80^d^	
≤ 50%	Substrate assay									
	Acrosin target with gelatine substrate assay	1	0.26	0.97	9.64	0.76	–	12.63	–	
= 0%	Fluorometry									
	PAb-acrosin	1	0.63	0.92	9.23	0.39	–	23.87	–	
	PAb-hyaluronidase	1	0.23	0.90	2.29	0.86	–	2.68	–	
	MAb 4D4-proacrosin	1	0.40	0.96	10.00	0.63	–	16.00	0.71^d^	
	Spectrophotometry									
	Spectrophotometry/BAPNA	4	0.78 (0.38–0.95)	0.63 (0.40–0.81)	2.11 (1.25–3.55)	0.35 (0.11–1.18)	1.78 (0.29–3.27)	5.94 (1.34–26.34)	0.74 (0.70–0.78)	
	Accu-sperm Spectrophotometry/BAPNA	1	0.61	0.84	3.90	0.46	–	8.4	–	

*SEN* sensitivity, *SPE* specificity, *PLR* positive likelihood ratio, *NLR* negative likelihood ratio, *DS* diagnostic score, *DOR* diagnostic odds ratio, *AUC* area under the summary receiver operating characteristic curve, *CI* confidence interval, *NC* not compared

^a^Comparison between subgroups with the Q test for heterogeneity

^b^Converted number = actual number × 2. Studies were duplicated for numbers ≥ 2 and < 4, based on the computation of bivariate mixed effects regression model for the lowest threshold of 4 studies

^c^Not calculated in original data or not reported

^d^Coordinates in scatter plots were converted with the Engauge digitizer to calculate AUC

After SEN analysis, two studies [8, 54] were identified as a source of heterogeneity when pooling Rs; however, after they were excluded, the correlation was unchanged (Rs = 0.38, 95% CI: 0.27–0.48, *p* <  0.001) and the heterogeneity while decreased was still significant (I^2^ = 67.1%, *p* = 0.001; Table 3). When SMD was pooled, four studies [3, 6, 50, 53] were found to contribute to this heterogeneity; when these were excluded, the correlation was unchanged (SMD = 0.75, 95% CI: 0.57–0.93, *p* <  0.001) but there was no obvious heterogeneity (I^2^ = 26.5%, *p* = 0.184; Table 4). When pooling diagnostic accuracy data, excluding one outlier [57] did not significantly change the overall results (SEN = 0.51, 95% CI: 0.39–0.62; SPE = 0.88, 95% CI: 0.79–0.94; PLR = 4.22, 95% CI: 2.42–7.36, NLR = 0.56, 95% CI: 0.45–0.69; DS = 2.02, 95% CI: 1.37–2.67; DOR = 7.53, 95% CI: 3.92–14.47; AUC = 0.73, 95% CI: 0.69–0.77; Table 7). Graphs of SROC curves generated before and after removing the outlier (Fig. 2e, f) indicated that the threshold effect applied to inter-study heterogeneity, since the spearman correlation coefficient between SEN and 1 − SPE was 0.685 (*p* = 0.014).

In the subgroup analysis (Table 5–7), datasets were stratified according to geographic area, sperm storage method, sperm preparation method, and FR cut-off value combined with AE assay method. The diagnostic performance in Asia (Rs = 0.24, 95% CI: 0.05–0.42, *p* = 0.013; SMD = 0.50, 95% CI: 0.15–0.85, *p* = 0.006) was inferior to that in North America (Rs = 0.54, 95% CI: 0.43–0.65, *p* <  0.001; SMD = 0.81, 95% CI: 0.39–1.22, *p* <  0.001) and Europe (Rs = 0.46, 95% CI: 0.37–0.54, *p* <  0.001; SMD = 0.92, 95% CI: 0.48–1.36, *p* <  0.001; comparison between subgroups [*p*] <  0.05). Cryopreserved spermatozoa (SMD = 0.20, 95% CI: − 0.11–0.52, *p* = 0.204; SEN = 0.51; SPE = 0.51; DOR = 1.12) were inferior to fresh spermatozoa (SMD = 0.89, 95% CI: 0.63–1.15, *p* <  0.001; SEN = 0.60, 95% CI: 0.41–0.76; SPE = 0.87, 95% CI: 0.77–0.93; DOR = 9.99, 95% CI: 6.05–16.49; comparison between subgroups [*p*] <  0.001). Sperm preparation yielded inferior results as compared to no preparation (Rs = 0.42, 95% CI: 0.30–0.55, *p* <  0.001; SMD = 0.82, 95% CI: 0.50–1.13, *p* <  0.001; SEN = 0.72, 95% CI: 0.50–0.87; SPE = 0.80, 95% CI: 0.59–0.92; DOR = 10.56, 95% CI: 5.51–20.26; comparison between subgroups [*p*] <  0.05); spermatozoa after swim up were scarcely irrelevant (Rs = 0.27, 95% CI: 0.01–0.53, *p* = 0.044); and there was no correlation for spermatozoa after a discontinuous gradient (SMD = 1.07, 95% CI: − 0.10–2.25, *p* = 0.074).

AE levels determined by fluorometry—including pAb-acrosin (SMD = 1.68, 95% CI: 1.29–2.07, *p* <  0.001), pAb-hyaluronidase (SMD = 0.48, 95% CI: 0.13–0.82, *p* = 0.007), and mAb 4D4-proacrosin (Rs = 0.49, 95% CI: 0.27–0.70, *p* <  0.001; SMD = 0.72, 95% CI: 0.32–1.13, *p* <  0.001)—were positively correlated with FR. For predicting TFF, the pAb-acrosin assay with a cut-off value of < 60% for normal fluorescence scores (SEN = 0.63, SPE = 0.92, DOR = 23.87); pAb-hyaluronidase assay with a cut-off value of < 80% for normal fluorescence scores (SEN = 0.23, SPE = 0.90, DOR = 2.68); and mAb 4D4-proacrosin assay with a cut-off value of ≤50% for the normal acrosomal principal region (SEN = 0.40, SPE = 0.96, DOR = 16.00) and low SEN and high SPE were adopted.

AE levels determined by spectrophotometry—including spectrophotometry/BAPNA (Rs = 0.44, 95% CI: 0.35–0.54, *p* <  0.001), Accu-Sperm spectrophotometry/BAPNA (SMD = 0.70, 95% CI: 0.41–0.99, *p* <  0.001)—were positively correlated with FR, but this did not apply to spectrophotometry/BAEE (SMD = − 0.04, 95% CI: − 0.72–0.64, *p* = 0.908). The spectrophotometry/BAPNA assay predicted an FR <  50%, with pooled low SEN (0.63, 95% CI: 0.48–0.76) and moderate SPE (0.87, 95% CI: 0.60–0.97) and DOR = 11.68 (95% CI: 3.47–39.36). Specifically, low SEN and high SPE and moderate SEN and low SPE were associated with cut-off values of 25 μIU/10^6^ spermatozoa (SEN = 0.51, SPE = 0.97, DOR = 33.78) [58] and 18 μIU/10^6^ spermatozoa (SEN = 0.76, SPE = 0.63, DOR = 5.42) [55]. For predicting TFF, the spectrophotometry/BAPNA assay was adopted with pooled moderate SEN and low SPE (SEN = 0.78, 95% CI: 0.38–0.95; SPE = 0.63, 95% CI: 0.40–0.81; DOR = 5.94, 95% CI: 1.34–26.34). Specifically, moderate SEN and SPE, high SEN and low SPE, low SEN and moderate SPE, and low SEN and SPE were obtained for cut-off values of 25 μIU/10^6^ spermatozoa (SEN = 0.78, SPE = 0.73, DOR = 9.50) [6], 54μIU/10^6^ spermatozoa (SEN = 1.00, SPE = 0.36, DOR = 23.31) [57], and 30 μIU/10^6^ spermatozoa (SEN = 0.53, SPE = 0.86, DOR = 7.27) [3], and 15 μIU/μg DNA (SEN = 0.51, SPE = 0.51, DOR = 1.12) [53]. The Accu-Sperm spectrophotometry/BAPNA assay with a cut-off value of < 4.5 for the acrosin activity index was adopted with low SEN and moderate SPE (SEN = 0.61, SPE = 0.84, DOR = 8.40).

AE levels determined by substrate assays—including acrosin target with gelatine substrate assay (Rs = 0.43, 95% CI: 0.30–0.56, *p* <  0.001), and hyaluronidase target with agar/hyaluronic acid mixture substrate assay (Rs = 0.54, 95% CI: 0.43–0.65, *p* <  0.001) —were positively correlated with FR. For predicting an FR of ≤50% or <  50%, the acrosin target with gelatine substrate assay with a cut-off value of < 6 for acrosin activity index or <  60% for halo formation rate showed low SEN and high SPE (SEN = 0.26, SPE = 0.97, DOR = 12.63; SEN = 0.50, SPE =0.93, DOR = 13.00, respectively).

The included studies were distributed symmetrically without obvious publication bias (Deeks’ funnel plot [*p*] = 0.53, Fig. 4b).

#### Spontaneous AR assay

Rs was extracted from 3 articles that included a total of 181 infertile couples. The spontaneous AR% and FRs that were higher and lower than the respective cut-off values were extracted from 9 articles, which included a total of 602 infertile couples. Of the 10 datasets analyzed, one used two AR assay methods [37]. Binary accuracy data were extracted from only 3 articles as 2 × 2 tables; the diagnostic summary measures were not pooled, based on the computation of bivariate mixed effects regression model for the lowest threshold of 4 studies (Table 3).

**Fig. 3:**
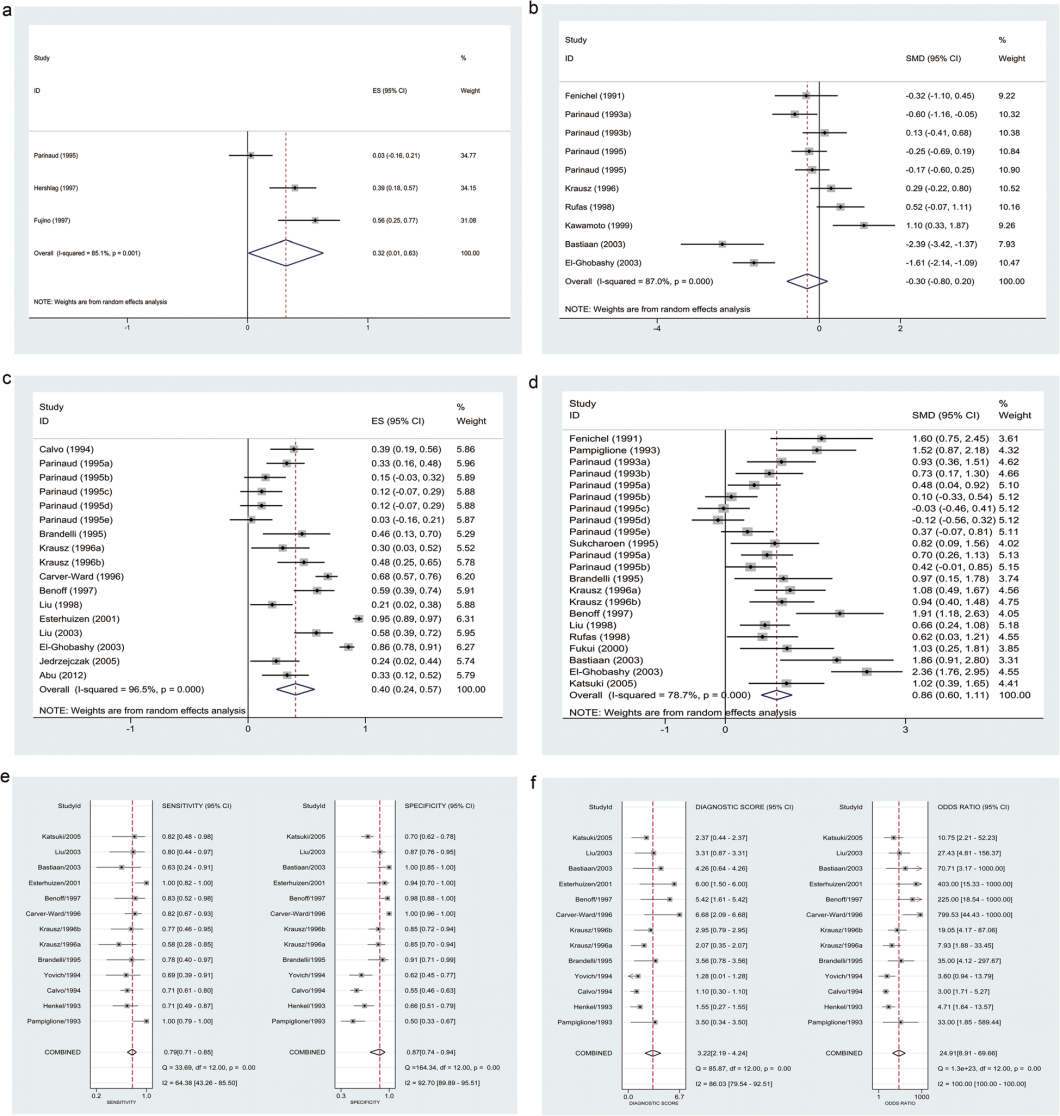
**a, b** Forest plots of Rs (**a**) and SMD (**b**) for spontaneous AR assay. Spontaneous AR% were weakly correlated with FR (pooled Rs = 0.32). However, the higher spontaneous AR% was not obtained for higher as compared to lower FRs when pooling SMD (pooled SMD = − 0.30). **c–f** Forest plots of Rs (**c**), SMD (**d**), SEN and SPE (**e**), DS and DOR (**f**) for induced AR assay. Induced AR% were positively correlated with FR (pooled Rs = 0.40). Higher induced AR% was obtained for higher as compared to lower FRs (pooled SMD = 0.86). Lower induced AR% was predictive of lower FR with moderate SEN, SPE (pooled SEN = 0.79; SPE = 0.87), and discriminant effect (pooled DS = 3.22; DOR = 24.91). *AR* acrosome reaction, *Rs* spearman correlation coefficient, *SMD* standardized mean difference, *SEN* sensitivity, SPE specificity, *DS* diagnostic score, *DOR* diagnostic odds ratio

**Fig. 4:**
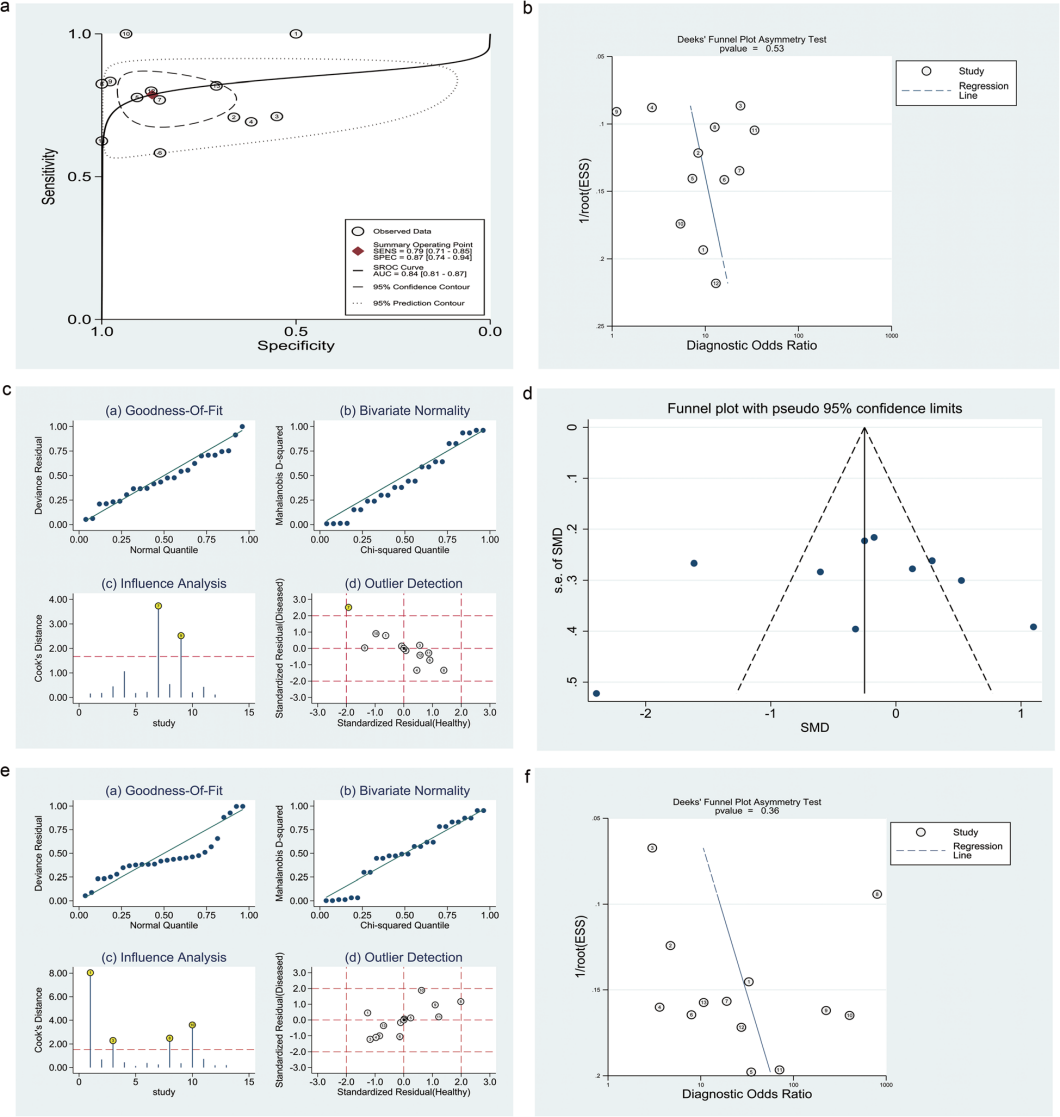
**a** Graph of SROC curve for induced AR assay. Lower induced AR% was predictive of lower FR with moderate accuracy (AUC = 0.84). **c, e** Sensitivity analysis plots for spontaneous (**c**) and induced AR assay (**e**). The goodness-of-fit and bivariate normality analyses showed that the bivariate model was moderately robust. The influence analysis and outlier detection identified one outlier for spontaneous AR assay, but there was no outlier was identified for induced AR assay. **b, d, f** Funnel plots for AE assay (**b**), spontaneous (**d**) and induced assay (**f**). The studies were distributed symmetrically without obvious publication bias in three funnel plots

According to a random-effects model, spontaneous AR% was weakly correlated with FR (Rs = 0.32, 95% CI: 0.01–0.63, *p* = 0.045; Fig. 3a), with notable heterogeneity (I^2^ = 85.1%, *p* = 0.001). However, the higher spontaneous AR% was not obtained for higher as compared to lower FRs when pooling SMD (SMD = − 0.30, 95% CI: –0.80–0.20, *p* = 0.245; Fig. 3b), with notable heterogeneity (I^2^ = 87.0%, *p <* 0.001). After SEN analysis, three studies [29, 33, 63] were identified as a source of heterogeneity; after they were excluded, the irrelevance was unchanged (SMD = − 0.06, 95% CI: –0.33–0.22, *p* <  0.001) but the heterogeneity significantly decreased (I^2^ = 46.2%, *p* = 0.084). The included studies were distributed symmetrically without obvious publication bias (Egger’s test [*p*] = 0.713, Fig. 4d).

#### Induced AR assay

Rs was extracted from 12 articles that included a total of 917 infertile couples. A total of 17 datasets were analyzed, including one article each that used five [41] and two [39] AR triggers. Induced AR% and FRs that were higher and lower than the respective cut-off values were extracted from 15 articles, which included a total of 1033 infertile couples. Of the 22 datasets analyzed, one used two AR assay methods [37], another reported five AR triggers [41], and two also mentioned different triggers [39, 42]. Binary accuracy data from 953 infertile couples were extracted from 12 articles as 2 × 2 tables. We analyzed the 13 datasets, including one paper that used two triggers [39] (Table 4).

According to a random-effects model, induced AR% were positively correlated with FR (Rs = 0.40, 95% CI: 0.24–0.57, *p* <  0.001; Fig. 3c, Table 8), albeit with notable heterogeneity (I^2^ = 96.5%, *p* <  0.001). Higher induced AR% was obtained for higher as compared to lower FRs (SMD = 0.86, 95% CI: 0.60–1.11, *p* <  0.001; Fig. 3d, Table 9). The bivariate mixed effects regression model predicted lower FR for lower induced AR% with pooled moderate SEN/SPE (SEN = 0.79, 95% CI: 0.71–0.85; SPE = 0.87, 95% CI: 0.74–0.94; Fig. 3e, Table 10), discriminant effect (PLR = 6.08, 95% CI: 2.77–13.36; NLR = 0.24, 95% CI: 0.17–0.35; DS = 3.22, 95% CI: 2.19–4.24; DOR = 24.91, 95% CI: 8.91–69.66; Fig. 3f, Table 10), and accuracy (AUC = 0.84, 95% CI: 0.81–0.87, Fig. 4a, Table 10). The Fagan nomogram showed that lower AE levels could be used to predict lower FR when the pre-test probability was 27%, with a post-test probability of 69%.

**Table 8:** Summary of Rs values for induced AR assay

Subgroup	N	R_s_ (95% CI)	Test(s) of heterogeneity		Significance test(s) of R_s_ = 0		*P* ^a^
			I^2^ (%)	*P*	Z	*P*	
Overall	17	0.40 (0.24–0.57)	96.5	0.000	4.87	0.000	
Three experiments excluded	14	0.36 (0.24–0.47)	83.6	0.000	5.99	0.000	
Geographic area							
Europe	10	0.33 (0.11–0.55)	95.7	0.000	2.98	0.003	< 0.001
Oceania	2	0.40 (0.03–0.76)	89.0	0.003	2.11	0.035	
South America	1	0.46 (0.17–0.75)	–^b^	–	3.14	0.002	
Africa	2	0.65 (0.05–1.25)	97.1	0.000	2.12	0.034	
North America	2	0.49 (0.30–0.69)	57.8	0.124	4.95	0.000	
Preparation method							
No preparation	1	0.39 (0.20–0.58)	–	–	4.12	0.000	< 0.001
One preparation	15	0.41 (0.24–0.58)	96.7	0.000	4.63	0.000	
Swim up	6	0.65 (0.49–0.81)	93.0	0.000	8.07	0.000	< 0.001
Swim up/discontinuous gradient	2	0.61 (0.42–0.81)	50.9	0.153	6.07	0.000	
Discontinuous gradient	7	0.17 (0.10–0.25)	19.0	0.285	4.42	0.000	
Double preparation	1	0.33 (0.13–0.53)	–	–	3.28	0.001	
AR trigger							
Physiological trigger	8	0.49 (0.28–0.70)	96.8	0.000	4.51	0.000	< 0.001
HFF	3	0.46 (−0.03–0.95)	97.3	0.000	1.85	0.065	< 0.001
P	2	0.31(−0.01–0.63)	82.4	0.017	1.89	0.059	
ZP	3	0.63 (0.25–1.01)	96.0	0.000	3.22	0.001	
Nonphysiological trigger	9	0.33 (0.15–0.50)	89.0	0.000	3.68	0.000	
A23187	5	0.36 (0.13–0.58)	89.1	0.000	3.12	0.002	< 0.001
CAMP	1	0.12(−0.06–0.29)	–	–	1.26	0.206	
TPA	1	0.03 (−0.15–0.21)	–	–	0.29	0.773	
BSA-GlcNAc/A23187	1	0.46 (0.17–0.75)	–	–	3.14	0.002	
Mannose	1	0.59 (0.42–0.76)	–	–	6.68	0.000	
AR assay method							
Fluorescent labels	16	0.41 (0.25–0.58)	96.5	0.000	4.89	0.000	< 0.001
Direct immunofluorescence	15	0.40 (0.21–0.58)	96.7	0.000	4.22	0.000	0.224
With lection	10	0.53 (0.36–0.70)	94.9	0.000	6.08	0.000	< 0.001
FITC-PSA	7	0.55 (0.36–0.75)	95.7	0.000	5.57	0.000	< 0.001
FITC-PNA	2	0.40 (0.23–0.58)	18.3	0.269	4.51	0.000	
RITC-PSA	1	0.59 (0.42–0.76)	–	–	6.68	0.000	
With antibody							
FITC-GB24	5	0.15 (0.05–0.25)	40.3	0.152	2.93	0.003	
Indirect immunofluorescence							
Anti-CD46 antibody	1	0.68 (0.59–0.77)	–	–	14.14	0.000	
Triple stain	1	0.24 (0.03–0.45)	–	–	2.24	0.025	

*Rs* spearman correlation coefficient, *AR* acrosome reaction, *CI* confidence interval, *HFF* human follicle fluid, *P* progesterone, *ZP* zona pellucida, *CAMP* cyclic adenosine 3′-5′-phosphate analogue, *TPA* phorbol ester, *BSA-GlcNAc* Neoglycoproteins with N-acetylglucosamine residues, *FITC-PSA* fluorescein isothiocyanate-conjugated Pisurn sativum agglutimm, *FITC-PNA* fluorescein isothiocyanate*-*conjugated peanut agglutinin, *RITC-PSA* rhodamine-conjugated Pisurn sativum agglutimm

^a^Comparison between subgroups with the Q test for heterogeneity

^**b**^Not calculated

**Table 9:** Summary of SMD values for induced AR assay

Subgroup	N	SMD (95% CI)	Test(s) of heterogeneity		Significance test(s) of SMD = 0		*P* ^a^
			I^2^ (%)	*P*	Z	*P*	
Overall	22	0.86 (0.60–1.11)	78.7	0.000	6.55	0.000	
Four experiments excluded	18	0.71 (0.52–0.90)	54.8	0.003	7.25	0.000	
Geographic area							
Europe	15	0.76 (0.44–1.07)	82.1	0.000	4.68	0.000	0.001
Oceania	1	0.66 (0.24–1.08)	–^b^	–	3.10	0.002	
South America	1	0.97 (0.15–1.78)	–	–	2.33	0.020	
Africa	1	1.86 (0.91–2.80)	–	–	4.46	0.000	
North America	1	1.91 (1.18–2.63)	–	–	5.14	0.000	
Asia	3	0.86 (0.48–1.24)	0.0	0.589	4.46	0.000	
Preparation method							
No preparation	0						
One preparation	22						
Swim up	8	1.51 (1.13–1.89)	61.3	0.012	7.75	0.000	< 0.001
Swim up/discontinuous gradient	1	0.97 (0.15–1.78)	–	–	4.79	0.000	
Discontinuous gradient	13	0.47 (0.28–0.66)	48.2	0.027	2.33	0.020	
Double preparation	0						
AR trigger							
Physiological trigger	7	0.95 (0.29–1.61)	89.1	0.000	2.81	0.005	0.92
HFF	3	0.97 (−0.42–2.37)	95.1	0.000	1.37	0.172	0.029
P	3	0.65 (0.01–1.28)	73.4	0.023	1.99	0.047	
ZP	1	1.86 (0.91–2.80)	–	–	3.85	0.000	
Nonphysiological trigger	15	0.81 (0.56–1.06)	67.6	0.000	6.36	0.000	
A23187	10	0.87 (0.66–1.08)	26.1	0.204	8.30	0.000	< 0.001
CAMP	1	−0.12 (−0.56–0.32)	–	–	0.54	0.588	
TPA	2	0.40 (0.09–0.70)	0.0	0.866	2.54	0.011	
BSA-GlcNAc/A23187	1	0.97 (0.15–1.78)	–	–	2.33	0.020	
Mannose	1	1.91 (1.18–2.63)	–	–	5.14	0.000	
AR assay method							
Fluorescent labels	21	0.82 (0.57–1.08)	78.3	0.000	6.26	0.000	0.012
Direct immunofluorescence	19	0.80 (0.52–1.07)	79.4	0.000	5.72	0.000	0.16
With lection	12	1.16 (0.84–1.47)	66.3	0.001	7.17	0.000	< 0.001
FITC-PSA	7	1.19 (0.68–1.71)	77.5	0.000	4.53	0.000	0.060
FITC-PNA	4	0.96 (0.66–1.25)	0.0	0.961	6.27	0.000	
RITC-PSA	1	1.91 (1.18–2.63)	–	–	7.17	0.000	
With antibody	7	0.28 (0.06–0.50)	44.2	0.097	2.45	0.014	
FITC-GB24							
Indirect immunofluorescence	2	1.11 (0.26–1.95)	64.2	0.094	2.57	0.010	
GB24 antibody							
Triple stain	1	1.52 (0.87–2.18)	78.7	0.000	4.57	0.000	

*Rs* spearman correlation coefficient, *AR* acrosome reaction, *CI* confidence interval, *HFF* human follicle fluid, *P* progesterone, *ZP* zona pellucida, *CAMP* cyclic adenosine 3′-5′-phosphate analogue, *TPA* phorbol ester, *BSA-GlcNAc* Neoglycoproteins with N-acetylglucosamine residues, *FITC-PSA* fluorescein isothiocyanate-conjugated Pisurn sativum agglutimm, *FITC-PNA* fluorescein isothiocyanate*-*conjugated peanut agglutinin, *RITC-PSA* rhodamine-conjugated Pisurn sativum agglutimm, FITC-GB24, fluorescein isothiocyanate*-*GB24

^a^Comparison between subgroups with the Q test for heterogeneity

^b^Not calculated

**Table 10:** Summary of Summary of SEN, SPE, PLR, NLR, DS, DOR, and AUC values for induced AR assay

Subgroup	N	SEN (95% CI)	SPE (95% CI)	PLR (95% CI)	NLR (95% CI)	DS (95% CI)	DOR (95% CI)	AUC (95% CI)	*P* ^a^
Overall	13	0.79 (0.71–0.85)	0.87 (0.74–0.94)	6.08 (2.77–13.36)	0.24 (0.17–0.35)	3.22 (2.19–4.24)	24.91 (8.91–69.66)	0.84 (0.81–0.87)	
Geographic area									
Europe	5	0.80 (0.66–0.89)	0.86 (0.56–0.97)	5.80 (1.46–23.10)	0.23 (0.13–0.43)	3.21 (1.51–4.91)	24.81 (4.53–135.75)	0.86 (0.83–0.89)	NC
Oceania	4^b^	0.75 (0.59–0.86)	0.77 (0.61–0.87)	3.22 (1.70–6.08)	0.33 (0.18–0.61)	2.28 (1.11–3.45)	9.78 (3.04–31.47)	0.80 (0.77–0.84)	
South America	1	0.78	0.91	8.56	0.24	−^c^	35.00	–	
Africa	4^b^	0.94 (0.44–1.00)	0.98 (0.85–1.00)	52.54 (5.96–462.88)	0.06 (0.00–1.00)	6.76 (3.74–9.77)	861.19 (42.25–17,552.00)	0.99 (0.98–1.00)	
North America	4^b^	0.77 (0.69–0.84)	0.87 (0.64–0.96)	6.11 (1.79–20.94)	0.26 (0.16–0.41)	3.17 (1.54–4.79)	23.70 (4.68–120.00)	0.83 (0.79–0.86)	
Asia	1	0.82	0.70	2.77	0.26	–	10.75	–	
Preparation method									
No preparation	1	0.71	0.55	1.58	0.53	–	3.00	–	NC
One preparation	11	0.82 (0.73–0.88)	0.87 (0.73–0.94)	6.10 (2.84–13.09)	0.21 (0.14–0.33)	3.36 (2.32–4.39)	28.70 (10.21–80.65)	0.88 (0.85–0.91)	
Double preparation	1	0.63	1.00	–	0.40	–	–	–	
AR trigger									
Physiological trigger	10^b^	0.82 (0.73–0.88)	0.88 (0.76–0.94)	6.78 (3.10–14.83)	0.20 (0.13–0.33)	3.50 (2.35–4.65)	32.26 (10.53–105.06)	0.89 (0.86–0.91)	NC
Nonphysiological trigger	8	0.79 (0.70–0.85)	0.86 (0.65–0.95)	5.70 (1.98–16.39)	0.25 (0.16–0.37)	3.14 (1.82–4.46)	23.10 (6.16–81.56)	0.82 (0.78–0.85)	
AR assay method									
Fluorescent labels	11	0.78 (0.71–0.84)	0.90 (0.78–0.96)	8.01 (3.24–19.82)	0.24 (0.18–0.34)	3.49 (2.35–4.64)	32.83 (10.44–103.21)	0.83 (0.80–0.86)	
Direct immunofluorescence	10	0.78 (0.70–0.85)	0.86 (0.74–0.93)	5.72 (2.81–11.63)	0.25 (0.17–0.37)	3.12 (2.11–4.14)	22.76 (8.23–62.95)	0.84 (0.81–0.87)	
FITC−PSA	7	0.81 (0.69–0.88)	0.83 (0.66–0.93)	4.80 (2.09–11.01)	0.23 (0.13–0.41)	3.02 (1.72–4.32)	20.51 (5.60–75.15)	0.87 (0.83–0.89)	
FITC−PNA	4^b^	0.68 (0.58–0.76)	0.85 (0.81–0.88)	4.55 (3.42–6.05)	0.38 (0.28–0.50)	2.49 (1.98–3.01)	12.10 (7.24–20.21)	0.85 (0.81–0.87)	
RITC−PSA	1	0.83	0.98	38.33	0.17	–	225.00	–	
Indirect immunofluorescence									
Anti−CD46 antibody	1	0.83	1.00	–	0.18	–	–	–	
Triple stain	4^b^	0.93 (0.76–0.98)	0.58 (0.52–0.64)	2.23 (1.96–2.54)	0.12 (0.03–0.43)	2.92 (1.59–4.25)	18.56 (4.91–70.16)	0.68 (0.64–0.72)	

*SEN* sensitivity, *SPE* specificity, *PLR* positive likelihood ratio, *NLR* negative likelihood ratio, *DS* diagnostic score, *DOR* diagnostic odds ratio, *AUC* area under the summary receiver operating characteristic curve, *CI* confidence interval, *AR* acrosome reaction, *FITC-PSA* fluorescein isothiocyanate-conjugated Pisurn sativum agglutimm, *FITC-PNA* fluorescein isothiocyanate*-*conjugated peanut agglutinin, *RITC-PSA* rhodamine-conjugated Pisurn sativum agglutimm, *NC* not compared

^a^Comparison between subgroups with the Q test for heterogeneity

^b^Converted number = actual number × 2. Studies were duplicated for numbers ≥ 2 and < 4, based on the computation of bivariate mixed effects regression model for the lowest threshold of 4 studies

^c^Not calculated in original data or not reported

After SEN analysis, seven studies (when pooling Rs: 3; when pooling SMD: 4) were identified as a source of heterogeneity; however, after they were excluded, the correlation was unchanged (Rs = 0.36, 95% CI: 0.24–0.47, *p* <  0.001; SMD = 0.71, 95% CI: 0.52–0.90, respectively) and the heterogeneity while decreased was still significant (I^2^ = 83.6%, *p* <  0.001; I^2^ = 54.8%, *p =* 0.003, respectively). There was no outlier was identified when pooling diagnostic accuracy data (Fig. 4e). Graphs of SROC curves generated indicated that the threshold effect did not apply to inter-study heterogeneity (*r* = − 0.146, *p* = 0.634; Fig. 4a).

In the subgroup analysis, datasets were stratified according to geographic area, sperm preparation method, AR trigger, and AR assay method (Tables 8, 9 and 10). The diagnostic performance in the other areas (Europe [Rs = 0.33, 95% CI: 0.11–0.55, *p* = 0.003; pooled moderate SEN = 0.80, 95% CI: 0.66–0.89; moderate SPE = 0.86, 95% CI: 0.56–0.97], Oceania [Rs = 0.40, 95% CI: 0.03–0.76, *p* = 0.035; pooled moderate SEN = 0.75, 95% CI: 0.59–0.86; moderate SPE = 0.77, 95% CI: 0.61–0.87], South America [Rs = 0.46, 95% CI: 0.17–0.75, *p* = 0.002; moderate SEN = 0.78, high SPE = 0.91], Asia [moderate SEN = 0.82, moderate SPE = 0.70], and North America [Rs = 0.49, 95% CI: 0.30–0.69, *p* < 0.001; pooled moderate SEN = 0.77, 95% CI: 0.69–0.84, moderate SPE = 0.87, 95% CI: 0.64–0.96) was inferior to that in Africa (Rs = 0.65, 95% CI: 0.05–1.25, *p* = 0.034; pooled high SEN = 0.94, 95% CI: 0.44–1.00, high SPE = 0.98, 95% CI: 0.85–1.00; comparison between subgroups [*p*] < 0.01).

No preparation (Rs = 0.39, 95% CI: 0.20–0.58, *p* < 0.001; moderate SEN = 0.71, low SPE = 0.55) or double preparation (Rs = 0.33, 95% CI: 0.13–0.53, *p* = 0.001; low SEN = 0.63, high SPE = 1.00) yielded inferior results as compared to one preparation (Rs = 0.41, 95% CI: 0.24–0.58, *p* < 0.001; pooled moderate SEN = 0.82, 95% CI: 0.73–0.88, moderate SPE = 0.87, 95% CI: 0.73–0.94; comparison between subgroups [*p*] < 0.001); discontinuous gradient (Rs = 0.17, 95% CI: 0.10–0.25, *p* < 0.001; SMD = 0.47, 95% CI: 0.28–0.66, *p* = 0.02) was inferior to swim up (Rs =0.65, 95% CI: 0.49–0.81, *p* < 0.001; SMD = 1.51, 95% CI: 1.13–1.89, *p* < 0.001; comparison between subgroups [*p*] < 0.001).

Nonphysiological triggers (SMD = 0.81, 95% CI: 0.56–1.06, *p* < 0.001; moderate SEN = 0.79, 95% CI: 0.70–0.85; pooled moderate SPE = 0.86, 95% CI: 0.65–0.95) did not differ from physiological triggers (SMD = 0.95, 95% CI: 0.29–1.61, *p =* 0.005; pooled moderate SEN = 0.82, 95% CI: 0.73–0.88; moderate SPE = 0.88, 95% CI: 0.76–0.94; comparison between subgroups [*p*] = 0.92) in general; ZP (Rs = 0.63, 95% CI: 0.25–1.01, *p* = 0.001; SMD = 1.86, 95% CI: 0.91–2.80, *p* < 0.001;) or mannose (Rs = 0.59, 95% CI: 0.42–0.76, *p* < 0.001; SMD = 1.91, 95% CI: 1.18–2.63, *p* < 0.001) was superior to other physiological (comparison between subgroups [*p*] < 0.05) or nonphysiological triggers (A23187 [Rs = 0.36, 95% CI: 0.13–0.58, *p* = 0.002; SMD = 0.87, 95% CI: 0.66–1.08, *p* < 0.001], BSA-GlcNAc [Rs = 0.46, 95% CI: 0.17–0.75, *p* = 0.002; SMD = 0.97, 95% CI: 0.15–1.78, *p* = 0.02]; comparison between subgroups [*p*] < 0.001); and there was no correlation for HFF (Rs = 0.46, 95% CI: − 0.03–0.95, *p* = 0.065; SMD = 0.97, 95% CI: − 0.42 − 2.37, *p* = 0.172), P (Rs = 0.31, 95% CI: − 0.01 − 0.63, *p* = 0.059), CAMP (Rs = 0.12, 95% CI: − 0.06 − 0.29, *p* = 0.206; SMD = − 0.12, 95% CI: − 0.56 − 0.32, *p* = 0.588) and TPA (Rs =0.03, 95% CI: -0.15–0.21, *p* = 0.773).

The diagnostic performance of fluorescent labels (Rs = 0.41, 95% CI: 0.25–0.58, *p* < 0.001; SMD = 0.82, 95% CI: 0.57–1.08, *p* < 0.001) did not differ from that of triple stain (Rs = 0.24, 95% CI: 0.03–0.45; SMD = 1.52, 95% CI: 0.87–2.18); Lower induced AR% determined by fluorescent labels or triple stain was used for predicting lower FR with pooled moderate SEN/high SPE (SEN = 0.78, 95% CI: 0.71–0.84; SPE = 0.90, 95% CI: 0.78–0.96) or pooled high SEN/low SPE (SEN = 0.93, 95% CI: 0.76–0.98; SPE = 0.58, 95% CI: 0.52–0.64). The diagnostic performance of direct immunofluorescence (Rs = 0.40, 95% CI: 0.21–0.58; SMD = 0.80, 95% CI: 0.52–1.07) did not differ from that of indirect immunofluorescence (Anti-CD46 antibdy [Rs = 0.68, 95% CI: 0.59–0.77, *p* < 0.001], GB24 antibody [SMD = 1.11, 95% CI: 0.26–1.95, *p* = 0.01]; comparison between subgroups [*p*] >  0.05); direct immunofluorescence with antibody (FITC-GB24: Rs = 0.15, 95% CI: 0.05–0.25, *p* = 0.003; SMD = 0.28, 95% CI: 0.06–0.50, *p* = 0.014) was inferior to direct immunofluorescence with lectin (Rs = 0.53, 95% CI: 0.36–0.70, *p* < 0.001; SMD = 1.16, 95% CI: 0.84–1.47, *p* < 0.001; comparison between subgroups < 0.001); there is no significant difference between lectins (FITC-PSA [SMD = 1.19, 95% CI: 0.68–1.71, *p* < 0.001], FITC-PNA [SMD = 0.96, 5% CI: 0.66–1.25, *p* < 0.001], and RITC-PSA [SMD = 1.91, 95% CI: 1.18–2.63, *p* < 0.001]; comparison between subgroups [*p*] = 0.06). Specifically, moderate SEN/moderate SPE (SEN = 0.81, 95% CI: 0.69–0.88; SPE = 0.83, 95% CI: 0.66–0.93), pooled low SEN/moderate SPE (SEN = 0.68, 95% CI: 0.58–0.76; SPE = 0.85, 95% CI: 0.81–0.88, and moderate SEN/high SPE (SEN = 0.83, SPE = 0.98) were obtained for FITC-PSA, FITC-PNA, and RITC-PSA.

The included studies were distributed symmetrically without obvious publication bias (Deeks’ funnel plot [p] = 0.36, Fig. 4f).

## Discussion

There are many functional assays that attempt to assess the fertilization capacity of spermatozoa based on hypoosmotic swelling, peroxidative damage, acrosome status, AEs, sperm chromatin, sperm-oocyte interaction, zona pellucida binding, and zona-free oocyte penetration [65]. However, their clinical utility for diagnosing male infertility is unclear. One reliable criterion for evaluating the diagnostic performance of assays is whether or not they can predict fertilization outcomes in IVF [3, 66, 67].

Our first results showed that AE (i.e., proacrosin and acrosin) levels determined by spectrophotometry/BAPNA were positively correlated with FR. However, lower AE levels were predictive of TFF with moderate SEN but with low SPE. In addition, a meta-analysis of published literature on similar topic was performed to further expand upon the findings. To the best of our knowledge, this meta-analysis is the first study to evaluate the association between acrosome function scoring—including AE levels and AR%—and FR and the diagnostic performance of acrosome function scoring. No attempt has been made here to correlate the scoring with conception rates because several other factors, such as the endometrial secretions, receptivity and systemic and local endocrine status, become significant after embryo transfer [54]. After validating the correlation with pooling Rs and SMD, lower AE levels or induced AR% was predictive of lower FR with moderate accuracy (AUC between 0.70–0.90); this was accompanied by low SEN/moderate SPE, moderate SEN/moderate SPE, respectively. A moderate SPE indicates that a male diagnosed as scoring -negative (i.e., higher than the AE cut-off value) has about 85% or greater probability of having a high FR (i.e., higher than the FR cut-off value). Fifteen percent of the patients with high AE levels and poor fertilization probably have defects other than impaired AEs [18]. For induced AR assay, the findings were in agreement with the results of Oehninger et al. [68], who reported that AR results were predictive of IVF rates, showing moderate accuracy, SEN and SPE. However, for AE levels, a low SEN indicates that a male diagnosed as AE-positive (i.e., low than the AE cut-off value) still has a 43% probability of having a high FR. The described first results are expected as proacrosin/acrosin is an important enzyme for fertilization. However, the SPE is low, probably because its action is dependent on structural and biochemical events which take place during capacitation and the acrosome reaction and it cannot be detected in its proper location (i.e., the acrosome) like fluorometry [3]. The other kinds of AEs, such as hyaluronidase, were not taken into consideration. Furthermore, the satisfying diagnostic performance was not obtained for assays in the meta-analysis, in spite of synthesizing multiple assay methods. This result in relatively low SEN might be attributed to other parameters of sperm function, such as good membrane integrity, normal chromatin decondensation, excellent ability of undergoing capacitation and hyperactivation, high inducibility of the acrosome reaction (AR), increased sperm-oolemma interaction, or mild peroxidative damage, low DNA fragmentation. However, it should be mentioned that the fertilization process is a multifactorial process where female factors, such as young woman, maturity of oocyte/spindle/zona pellucida, intactness of cumulus-oocyte complex, or good ability to modulate/restore sperm functions, may contribute to high fertilization [15, 16]. For spontaneous AR assay, a weak correlation was obtained when pooling Rs; however, after enlarging the sample size, there was no significant correlation between them when pooling SMD. The spontaneous AR assay was considered for the evaluation of the initial acrosome stability before ZP binding; a low percentage of spontaneous AR did not seem to influence sperm fertility may due to high heterogeneity of spermatozoa.

In addition, there was notable heterogeneity when pooling summary measures in the present meta-analysis. After SEN analysis, two studies were identified as a source of heterogeneity when pooling Rs for AE assay. One reported a linear correlation between AE and the percentage of cases with ≥70% fertilization achieved by IVF [8]. On the other hand, semen prepared by α-chymoytrypsin treatment was suitable for highly viscous semen [54]. When SMD was pooled, four studies [3, 6, 50, 53] were found to contribute to this heterogeneity. Two used cryopreserved spermatozoa to assay AE [50, 53]; one used spermatozoa without preparation [6] or spermatozoa subjected to a special discontinuous gradient (i.e., 1-ml fractions of 90%, 80%, and 50% Percoll in isotonic Ham’s-F10) [3] in IVF therapy. When pooling diagnostic accuracy data, one outlier may have affected inter-study heterogeneity, for which the highest AE cut-off value was obtained by the spectrophotometry/BAPNA assay (54 μIU/10^6^ spermatozoa) [57]. The sperm origin (fresh or cryopreserved), sperm preparation methods, FR cut-off values, and AE assay methods and cut-off values might contribute to inter-study heterogeneity. For spontaneous AR assay, three studies found to contribute to this heterogeneity when pooling SMD. Two used FITC-PSA to determine AR after incubation for 60 min in synthetic human tubal fluid (HTF) media [33, 63]; one used two-color fluorescence staining of FITC-PSA and anti-CD46 antibody (MH61) to assay acrosomal status after 4 h of incubation in mBWW/3.5% HSA media [29]. The sperm capacitation time, media, and assay methods might contribute to inter-study heterogeneity. For induced AR assay, seven studies [31, 33, 41, 43, 63] were identified as a source of heterogeneity. The inconsistencies among studies regarding capacitation time (range between 1 h and 24 h), sperm preparation methods (swim up or discontinuous gradient), AR triggers (physiological [HFF, P, ZP] or nonphysiological [TPA, CAMP, mannose]), as well as AR assay methods (FITC-PSA, RITC-PSA, FITC-GB24) methods might contribute to inter-study heterogeneity.

Furthermore, the subgroup analysis revealed that the correlation between AE levels and FR depended on geographic area, with Asia being inferior in this regard to North America and Europe, which may be explained by methodological quality. For example, two of three studies in Asia [9, 18] did not describe the inclusion criteria for patients undergoing IVF therapy, whereas only a minority of North American (i.e., three of seven) [6, 8, 19] and European (i.e., one in six articles) [3] studies did not report these criteria. In addition, two Asian studies [9, 57] did not clearly define the reference standard test (i.e., fertilization), which was only true for two North American [6, 50] and one European [54] study. Additionally, there may be racial differences that could possibly contribute, but this is unknown. The populations of certain areas of the world, such as in parts of North America, can be very heterogeneous as well and racial status cannot be assumed. In the sperm head, the organelle most affected by cryopreservation damage was the acrosome [69], suggesting that cryopreserved spermatozoa were inferior to fresh spermatozoa. Spermatozoa without preparation more closely reflected the population composition and fertility of the original ejaculate [6] and were superior to spermatozoa after swim up and a discontinuous gradient in terms of diagnostic performance. It was difficult to predict FR based on AE levels with high accuracy as well as SEN and SPE using any one assay method. The lower AE levels determined by fluorometry—including pAb-acrosin, pAb-hyaluronidase, and mAb 4D4-proacrosin—could predict TFF with low SEN and high SPE. Lower AE levels determined by the gelatine substrate assay could predict lower FR (i.e., FR ≤ 50% or <  50%) with low SEN and high SPE. As for the hyaluronidase target with agar/hyaluronic acid mixture substrate assay, the diagnostic performance was not evaluated because the described high SEN (0.91) and SPE (1.00) for predicting TFF in the text has contradiction with the calculated low SEN (0.54) and high SPE (1.00) from scatterplot of correlation between hyaluronidase activity and FR in the study by Abdul-Aziz et al. [19]. More studies are needed to determine its predictability. The spectrophotometry assay had an uncertain predictive value. Specifically, the lower AE levels determined by the most commonly used spectrophotometry/BAPNA assay could predict a FR <  70%, FR <  50%, or FR = 0%; this was accompanied by moderate SEN/moderate SPE, pooled low SEN/moderate SPE and moderate SEN/low SPE, respectively. This result also validated the finding from retrospective study. AE levels determined by Accu-Sperm spectrophotometry/BAPNA could predict TFF with low SEN and moderate SPE. However, the lower AE levels obtained by spectrophotometry/BAEE in one study were not correlated with TFF. Another study [10] that was not included in our analysis showed similar results by the same method (AE extraction with acid [i.e., pH = 2.8]) but did not reflect the actual levels of proacrosin converted to acrosin.

For induced AR assay, the diagnostic performance also showed regional effects; the Africa in this regard was superior to other areas, which may be explained by methodology or high inter-study heterogeneity in other certain areas. For example, all three studies [31, 35, 63] in Africa used the same sperm preparation method (swim up), trigger (ZP), and assay method (FITC-PSA) and clearly defined the reference standard test. Two of them executed the laboratory quality control for assay method by establishing intra- and interassay/technician coefficients of variations, but only one study in other area did [34]. The spermatozoa after one preparation—especially swim up—show better survival after incubation in capacitation media compared with no-prepared or double-prepared spermatozoa, which may explain its optimal diagnostic performance [70]. The nonphysiological triggers did not differ from physiological triggers in terms of diagnostic performance; the mannose maybe act as a substitute when lack of physiological triggers. Nevertheless, the use of human ZP, biologically active recombinant ZP3 or active, synthetic ZP3 peptides (or analogues) combined with a better understanding of the biochemistry of the carbohydrate–protein interactions that take place during gamete recognition, binding and induction of acrosomal exocytosis will undoubtedly help in their elaboration [68]. Finally, it was difficult to predict FR based on induced AR% with high accuracy as well as SEN and SPE using any one assay method. Multiple methods (i.e., indirect immunofluorescence, direct immunofluorescence with lection, and triple stain) may be combined to obtain high SEN and SPE.

In conventional IVF therapy, one of the major disappointments that infertile couples may encounter is the unexpected failure to achieve fertilization. Some researches using early rescue ICSI procedure performed 4–6 h post-insemination have described successful salvage of some total or near-total fertilization failure cycles [71, 72]. Therefore, it may provide more important clinic direction when the acrosome function assays were used for predicting TFF. For AE assay, lower AE levels determined by spectrophotometry/BAPNA, Accu-Sperm spectrophotometry/BAPNA, or fluorometry—including pAb-acrosin assay, pAb-hyaluronidase, and mAb 4D4-proacrosin—were used for predicting TFF, with moderate SEN/low SPE, low SEN/moderate SPE, or low SEN/high SPE. For induced AR assay, lower induced AR% determined by triple stain or direct immunofluorescence with lection—including FITC-PSA and FITC-PNA—was used for predicting TFF, with high SEN/low SPE and moderate SEN/moderate SPE. Based on optimal diagnostic performance, a two-method assay using AE levels determined by pAb-acrosin assay and induced AR% determined by triple stain can be recommended for assessing acrosome function and predicting TFF. Two-method assay will reveal four types of detection results: AE levels-postive (< 60% for normal fluorescence scores)/induced AR%-positive (< 31.3% for difference between induced AR minus the spontaneous AR results), AE levels-negative (≥ 60% for normal fluorescence scores)/induced AR%-negative (≥ 31.3% for difference between induced AR minus the spontaneous AR results), AE levels-positive/induced AR%-negative, and AE levels-negative/induced AR%-positive. The early rescue ICSI procedure should be recommended for the patients diagnosed as AE levels-postive/induced AR%-positive, for which has a higher chance of TFF, or patients with high-risk factors—such as unexplained infertility or primary infertility with longer infertility duration—and with conflicting diagnosis (i.e., AE levels-postive/induced AR%-negative or AE levels-negative/induced AR%-positive). The conventional IVF therapy should be recommended for the patients diagnosed as AE levels-negative/induced AR%-negative, for which has a higher chance of fertilization success, or patients with conflicting diagnosis but without high-risk factors.

Our cohort study has several limitations: First, our dataset was collected retrospectively from a single center in a single geographic area and AE was determined by a single spectrophotometric method. Second, the sample size was not large and only FR was the primary fertilization outcome. The meta-analysis results should be considered in the context of their strengths and limitations. The advantages were as follows: the pooling of multiple summary measures; SEN and subgroup analyses to identify sources of heterogeneity; and low publication bias, which confirmed the reliability of the results. Nonetheless, there were some limitations such as no available RCT; the inclusion of old articles (published between 1988 and 2014) and studies with high heterogeneity; and the omission of some AE assay methods, including acrosin/proacrosin/acrosin inhibitor [12] or hyaluronidase [13] target with BAEE substrate assay, hyaluronidase target with cytochemical [14] or hyaluronic acid substrate [2] assay; and acrosin target with western blotting [5] or RIA [20], for which articles were lacking.

## Conclusions

The results of our study demonstrate that the acrosome function assays used to predict FR with high SEN and SPE are deficient. A limited prediction was obtained for AE assays, even though multiple methods (i.e., fluorometry, spectrophotometry, substrate assays) may be combined. But for induced AR assay, multiple methods (i.e., indirect immunofluorescence, direct immunofluorescence with lection, and triple stain) may be combined to obtain high SEN and SPE. The diagnostic performance showed regional effects as well as an effect of the sperm preparation or assay method. New assays of acrosome function—such as ones utilizing a panel of monoclonal or polyclonal antibodies against acrosome-related proteins—should be developed as a supplement for a more accurate diagnosis of structural and functional defects in the sperm acrosome. In addition, although most fertility centers rather prefer ICSI than IVF as method of treatment for male-factor infertility couples, yet the pace of this decision-making process should slow down, considering the controversy in the potential safety about ICSI. More studies of multicenter, large-scale, careful design and synthesizing multiple sperm functional assays and oocyte quality assays are still needed in clinical settings to better predict fertilization outcome in IVF. The early rescue ICSI procedure should be recommended for the patients with a higher chance of fertilization failure, and the conventional IVF therapy should be recommended for the patients with a higher chance of fertilization success.

## Additional files

Additional file 1: Search strategy. (DOCX 17 kb)

Additional file 2: **Table S1.** Spearman correlation between fertilization rate and baseline characteristics, and AE result. (DOCX 15 kb)

Additional file 3: **Table S2.** Grouping of patients according to AE result. (DOCX 14 kb)

## Abbreviations

AE: Acrosomal enzyme
AUC: Area under the summary receiver operating characteristic curve
BAEE: *N*-benzoyl-l-arginine ethyl ester
BAPNA: *N*-α-benzoyl-dl-arginine-p-nitroanilide
BSA-GlcNAc: Neoglycoproteins with N-acetylglucosamine residues
CAMP: Cyclic adenosine 3′-5′-phosphate analogue
CI: Confidence interval
DBM: Dyes for bright-field microscopy
DOR: Diagnostic odds ratio
DS: Diagnostic score
FITC-Con A: Concanavalin A lectin
FITC-GB24: Fluorescein isothiocyanate-GB24
FITC-PNA: Fluorescein isothiocyanate-conjugated peanut agglutinin
FITC-PSA: Fluorescein isothiocyanate-conjugated Pisurn sativum agglutimm
FR: Fertilization rate
HFF: Human follicle fluid
ICSI: Intracytoplasmic sperm injection
IVF: in vitro fertilization
mAb 4D4-proacrosin: Monoclonal anti-proacrosin antibody
NLR: Negative likelihood ratio
P: Progesterone
pAb-acrosin: Polyclonal anti-acrosin antibody
pAb-hyaluronidase: Polyclonal anti-hyaluronidase antibody
PLR: Positive likelihood ratio
RIA: Radioimmunoassay
RITC-PSA: Rhodamine-conjugated Pisurn sativum agglutimm
RR: Risk ratio
Rs: Spearman correlation coefficient
SEN: Sensitivity
SMD: Standardized mean difference
SPE: Specificity
SROC: Summary receiver operating characteristic
TEM: Transmission electron microscopy
TFF: Total fertilization failure
TPA: Phorbol ester
TRITC-PSA: Tetramethylrhodamine-conjugated PSA
ZP: Zona pellucida

## References

[1] Vazquez-Levin MH, Furlong LI, Veaute CM, Ghiringhelli PD (2005). An overview of the proacrosin/acrosin system in human spermatozoa. Treballs la Soc Catalana Biol.

[2] Hirayama T, Hasegawa T, Hiroi M (1989). The measurement of hyaluronidase activity in human spermatozoa by substrate slide assay and its clinical application. Fertil Steril.

[3] Senn A, Germond M, De Grandi P (1992). Immunofluorescence study of actin, acrosin, dynein, tubulin and hyaluronidase and their impact on in-vitro fertilization. Hum Reprod.

[4] Albert M, Gallo JM, Escalier D, Parseghian N, Jouannet P, Schrevel J (1992). Unexplained in-vitro fertilization failure: implication of acrosomes with a small reacting region, as revealed by a monoclonal antibody. Hum Reprod.

[5] Howe SE, Grider SL, Lynch DM, Fink LM (1991). Antisperm antibody binding to human acrosin: a study of patients with unexplained infertility. Fertil Steril.

[6] Kennedy WP, Kaminski JM, Ven HH, Der V, Jeyendran RS, Reid DS (1989). A simple, clinical assay to evaluate the acrosin activity of human spermatozoa. J Androl.

[7] Tummon IS, Yuzpe AA, Daniel SAJ, Deutsch A (1991). Total acrosin activity correlates with fertility potential after fertilization in vitro. Fertil Steril.

[8] De Jonge CJ, Tarchala SM, Rawlins RG, Binor Z, Radwanska E (1993). Acrosin activity in human spermatozoa in relation to semen quality and in-vitro fertilization. Hum Reprod.

[9] Yang YS, Chen SU, Ho HN, Chen HF, Lien YR, Lin HR (1994). Acrosin activity of human sperm did not correlate with IVF. Arch Androl.

[10] Liu DY, Baker HWG (1990). Relationships between human sperm acrosin, acrosomes, morphology and fertilization in vitro. Hum Reprod.

[11] Schill WB (1974). Quantitative determination of acrosin activity in human spermatozoa. Fertil Steril.

[12] Goodpasture JC, Polakoski KL, Zaneveld LJD (1980). Acrosin, proacrosin, and acrosin inhibitor of human spermatozoa: extraction, quantitation, and stability. J Androl.

[13] Zaneveld LJD, Polakoski KL, Schumacher GFB (1973). Properties of acrosomal hyaluronidase from bull spermatozoa evidence for its similarity to testicular hyaluronidase. J Biol Chem.

[14] Joyce C, Jeyendran RS, Zaneveld LJD (1985). Release, extraction, and stability of hyaluronidase associated with human spermatozoa. Comparisons with the rabbit. J Androl.

[15] Henkel R, Maaß G, Bödeker R, Scheibelhut C, Stalf T, Mehnert C (2005). Sperm function and assisted reproduction technology. Reprod Med Biol.

[16] Henkel R, Müller C, Miska W, Schill W, Kleinstein J, Gips H (1995). Acrosin activity of human spermatozoa by means of a simple gelatinolytic technique: a method useful for IVF. J Androl.

[17] Liu DY, Baker HWG (1994). Andrology: disordered acrosome reaction of spermatozoa bound to the zona pellucida: a newly discovered sperm defect causing infertility with reduced sperm-zona pellucida penetration and reduced fertilization in vitro. Hum Reprod.

[18] Tavalaee M, Razavi S, Nasr-Esfahani MH (2007). Effects of sperm acrosomal integrity and protamine deficiency on in vitro fertilization and pregnancy rate. Int J Fertil Steril.

[19] Abdul-Aziz M, MacLusky NJ, Bhavnani BR, Casper RF (1995). Hyaluronidase activity in human semen: correlation with fertilization in vitro. Fertil Steril.

[20] Mohsenian M, Syner FN, Moghissi KS (1982). A study of sperm acrosin in patients with unexplained infertility. Fertil Steril.

[21] Cross NL, Meizel S (1989). Methods for evaluating the acrosomal status of mammalian sperm. Biol Reprod.

[22] Fujino Y, Ozaki K, Nakamura Y, Sun TT, Ueda K, Ozaki A (1997). Clinical application of a new stain to detect acrosome-reacted sperm for predicting polyspermic fertilization in IVF-ET. Arch Androl.

[23] Pampiglione JS, Tan S-L, Campbell S (1993). The use of the stimulated acrosome reaction test as a test of fertilizing ability in human spermatozoa. Fertil Steril.

[24] Jędrzejczak P, Pawelczyk L, Taszarek-Hauke G, Kotwicka M, Warchoł W, Kurpisz M (2015). Predictive value of selected sperm parameters for classical in vitro fertilization procedure of oocyte fertilization. Andrologia.

[25] Calvo L, Dennison-Lagos L, Banks SM, Dorfmann A, Thorsell LP, Bustillo M (1994). Arosome reaction inducibility predicts fertilization success at in-vitro fertilization. Hum Reprod.

[26] Yovich JM, Edirisinghe WR, Yovich JL (1994). Use of the acrosome reaction to ionophore challenge test in managing patients in an assisted reproduction program: a prospective, double-blind, randomized controlled study. Fertil Steril.

[27] Brandelli A, Miranda PV, Añón-Vazquez MG, Marin-Briggiler CI, Sanjurjo C, Gonzalez-Echeverria F (1995). A new predictive test for in-vitro fertilization based on the induction of sperm acrosome reaction by N-acetylglucosamine-neoglycoprotein. Hum Reprod.

[28] Liu DY, Baker HWG (1998). Calcium ionophore-induced acrosome reaction correlates with fertilization rates in vitro in patients with teratozoospermic semen. Hum Reprod.

[29] Kawamoto A, Ohashi K, Kishikawa H, Zhu L-Q, Azuma C, Murata Y (1999). Two-color fluorescence staining of lectin and anti-CD46 antibody to assess acrosomal status. Fertil Steril.

[30] Fukui K, Hori R, Yoshimoto I, Ochi H, Ito M (2000). Correlation between progesterone binding sites on human spermatozoa and in vitro fertilization outcome. Gynecol Obstet Investig.

[31] Esterhuizen AD, Franken DR, Lourens JGH, Rooyen LHV (2001). Clinical importance of zona pellucida-induced acrosome reaction and its predictive value for IVF. Hum Reprod.

[32] Liu DY, Baker HWG (2003). Disordered zona pellucida–induced acrosome reaction and failure of in vitro fertilization in patients with unexplained infertility. Fertil Steril.

[33] El-Ghobashy AA, WEST CR (2003). The human sperm head: a key for successful fertilization. J Androl.

[34] Katsuki T, Hara T, Ueda K, Tanaka J, Ohama K (2005). Prediction of outcomes of assisted reproduction treatment using the calcium ionophore-induced acrosome reaction. Hum Reprod.

[35] Abu DAH, Franken DR, Hoffman B, Henke R (2012). Sequential analysis of sperm functional aspects involved in fertilisation: a pilot study. Andrologia.

[36] Wiser A, Sachar S, Ghetler Y, Shulman A, Breitbart H (2014). Assessment of sperm hyperactivated motility and acrosome reaction can discriminate the use of spermatozoa for conventional in vitro fertilisation or intracytoplasmic sperm injection: preliminary results. Andrologia.

[37] Parinaud J, Labal B, Vieitez G, Richoilley G, Grandjean H (1993). Comparison between fluorescent peanut agglutinin lectin and GB24 antibody techniques for the assessment of acrosomal status. Hum Reprod.

[38] Sukcharoen N, Keith J, Irvine DS, Aitken RJ (1995). Predicting the fertilizing potential of human sperm suspensions in vitro: importance of sperm morphology and leukocyte contamination. Fertil Steril.

[39] Krausz C, Bonaccorsi L, Maggio P, Luconi M, Criscuoli L, Fuzzi B (1996). Two functional assays of sperm responsiveness to progesterone and their predictive values in in-vitro fertilization. Hum Reprod.

[40] Takahashi K, Sakoda R, Yamasaki H, Uchida A, Kazuo Y, Kitao M (1993). Evaluation of sperm fertilizing capacity using the determination of acrosome reaction. Asia-Oceania J Obstet Gynaecol.

[41] Parinaud J, Vieitez G, Moutaffian H, Richoilley G, Labal B (1995). Variations in spontaneous and induced acrosome reaction: correlations with semen parameters and in-vitro fertilization results. Hum Reprod.

[42] Parinaud J, Vieitez G, Moutaffian H, Richoilley G, Labal B (1995). Relevance of acrosome function in the evaluation of semen in vitro fertilizing ability. Fertil Steril.

[43] Benoff S, Hurley IR, Mandel FS, Paine T, Jacob A, Cooper GW (1997). Use of mannose ligands in IVF screens to mimic zona pellucidainduced acrosome reactions and predict fertilization success. Fertil Steril.

[44] Hershlag A, Paine T, Scholl GM, Rosenfeld D, Mandel FS, Zhu JZ (1997). Acrobeads test as a predictor of fertilization in vitro. Am J Reprod Immunol.

[45] Robertson L, Wolf DP, Tash JS (1988). Temporal changes in motility parameters related to acrosomal status: identification and characterization of populations of hyperactivated human sperm. Biol Reprod.

[46] Lee CY, Wong E, Hsu E, Huang ES (1993). Molecular identity of a sperm acrosome antigen recognized by HS-63 monoclonal antibody. J Reprod Immunol.

[47] Fénichel P, Donzeau M, Farahifar D, Basteris B, Ayraud N, Hsi B-L (1991). Dynamics of human sperm acrosome reaction: relation with in vitro fertilization. Fertil Steril.

[48] Carver-ward JA, Jaroudi KA, Hollanders JMG, Einspenner M (1996). High fertilization prediction by flow cytometric analysis of the CD46 antigen on the inner acrosomal membrane of spermatozoa. Hum Reprod.

[49] Lee MA, Trucco GS, Bechtol KB, Wummer N, Kopf GS, Blasco L (1987). Capacitation and acrosome reactions in human spermatozoa monitored by a chlortetracycline fluorescence assay. Fertil Steril.

[50] Kruger TF, Haque D, Acosta AA, Pleban P, Swanson RJ, Simmons KF (1988). Correlation between sperm morphology, acrosin, and fertilization in an IVF program. Arch Androl.

[51] Sofikitis NV, Miyagawa I, Zavos PM, Toda T, Iino A, Terakawa N (1994). Confocal scanning laser microscopy of morphometric human sperm parameters: correlation with acrosin profiles and fertilizing capacity. Fertil Steril.

[52] Romano R, Santucci R, Marrone V, Gabriele AR, Necozione S, Valenti M (1998). A prospective analysis of the accuracy of the TEST-yolk buffer enhanced hamster egg penetration test and acrosin activity in discriminating fertile from infertile males. Hum Reprod.

[53] Yie S, Baillie J, Younglai EV (1996). Acrosin activity in pelleted frozen sperm does not correlate with in vitro fertilization of oocytes. Andrologia.

[54] Sharma R, Hogg J, Bromham DR (1993). Is spermatozoan acrosin a predictor of fertilization and embryo quality in the human?. Fertil Steril.

[55] Menkveld R, Rhemrev JP, Franken DR, Vermeiden JP, Kruger TF (1996). Acrosomal morphology as a novel criterion for male fertility diagnosis: relation with acrosin activity, morphology (strict criteria), and fertilization in vitro. Fertil Steril.

[56] Langlois MR, Oorlynck L, Vandekerckhove F, Criel A, Bernard D, Blaton V (2005). Discrepancy between sperm acrosin activity and sperm morphology: significance for fertilization in vitro. Clin Chim Acta.

[57] Bartoov B, Reichart M, Eltes F, Lederman H, Kedem P (1994). Relation of human sperm acrosin activity and fertilization in vitro. Andrologia.

[58] Aghajanpour S, Ghaedi K, Salamian A, Deemeh MR, Tavalaee M, Moshtaghian J (2011). Quantitative expression of phospholipase C zeta, as an index to assess fertilization potential of a semen sample. Hum Reprod.

[59] Henkel R, Müller C, Miska W, Gips H, Schill WB (1993). Determination of the acrosome reaction in human spermatozoa is predictive of fertilization in vitro. Hum Reprod.

[60] Whiting PF, Rutjes AWS, Westwood ME, Mallett S, Deeks JJ, Reitsma JB (2011). QUADAS-2: a revised tool for the quality assessment of diagnostic accuracy studies. Ann Intern Med.

[61] Green S, Higgins JPT, Alderson P, Alderson P, Clarke M, Mulrow CD (2009). Cochrane handbook for systematic reviews of interventions.

[62] He Y, Liu H, Zheng H, Li L, Fu X, Liu J (2018). Effect of early cumulus cells removal and early rescue ICSI on pregnancy outcomes in high-risk patients of fertilization failure. Gynecol Endocrinol.

[63] Bastiaan HS, Windt ML, Menkveld R, Kruger TF, Oehninger S, Franken DR (2003). Relationship between zona pellucida-induced acrosome reaction, sperm morphology, sperm-zona pellucida binding and in vitro fertilization. Fertil Steril.

[64] Rufas O, Gilman A, Fisch B, Shalgi R (1998). Spontaneous and follicular fluid-induced acrosome reaction in sperm samples from in vitro fertilizing and nonfertilizing normozoospermic patients. J Assist Reprod Genet.

[65] World Health Organization, Department of Reproductive Health and Research (2010). WHO Laboratory Manual for the Examination and Processing of Human Semen.

[66] Liu DY, Du PY, Nayudu PL, Johnston WI, Baker HW (1988). The use of in vitro fertilization to evaluate putative tests of human sperm function. Fertil Steril.

[67] Acosta AA, Oehninger S, Morshedi M, Swanson RJ, Scott R, Irianni F (1989). Assisted reproduction in the diagnosis and treatment of the male factor. Obstet Gynecol Surv.

[68] Oehninger S, Franken DR, Sayed E, Barroso G, Kolm P (2000). Sperm function assays and their predictive value for fertilization outcome in IVF therapy: a meta-analysis. Hum Reprod Update.

[69] Gómez-Torres MJ, Medrano L, Romero A, Fernández-Colom PJ, Aizpurúa J (2017). Effectiveness of human spermatozoa biomarkers as indicators of structural damage during cryopreservation. Cryobiology.

[70] Thijssen A, Klerkx E, Huyser C, Bosmans E, Campo R, Ombelet W (2014). Influence of temperature and sperm preparation on the quality of spermatozoa. Reprod BioMed Online.

[71] Huang B, Li Z, Zhu L, Hu D, Liu Q, Zhu G (2014). Progesterone elevation on the day of HCG administration may affect rescue ICSI. Reprod Biomed.

[72] Li M, Wang H, Li W, Shi J (2017). Effect of normal sperm morphology rate (NSMR) on clinical outcomes for rescue-ICSI (R-ICSI) patients. Gynecol Endocrinol.

