# Supplementary material for: Human sperm acrosome function assays are predictive of fertilization rate in vitro: a retrospective cohort study and meta-analysis
Source: Reprod Biol Endocrinol. 2018 Aug 24;16:81. doi: 10.1186/s12958-018-0398-y (PMC6109296; doi:10.1186/s12958-018-0398-y)
Supplement: Supplementary file 1 — Search strategy. (DOCX 17 kb) [file 12958_2018_398_MOESM1_ESM.docx]

Search strategy

**AE assay**

The search strategy in Pubmed consisted of the following phrases:

(((Fertilization in Vitro[MeSH] OR In Vitro Fertilization[Title/Abstract] OR In Vitro Fertilizations[Title/Abstract] OR in-Vitro Fertilization[Title/Abstract] OR in-Vitro Fertilizations[Title/Abstract] OR Test-Tube Fertilization[Title/Abstract] OR Fertilization, Test-Tube[Title/Abstract] OR Fertilizations, Test-Tube[Title/Abstract] OR Test Tube Fertilization[Title/Abstract] OR Test-Tube Fertilizations[Title/Abstract] OR Fertilizations in Vitro[Title/Abstract] OR Test-Tube Baby[Title/Abstract] OR Babies, Test-Tube[Title/Abstract] OR Baby, Test-Tube[Title/Abstract] OR Test Tube Babies[Title/Abstract] OR Test-Tube Baby[Title/Abstract] OR Test tube baby technique[Title/Abstract] OR Test tube babies technique[Title/Abstract] OR Test tube ovum fertilization[Title/Abstract] OR artificial fertilization[Title/Abstract] OR ivf[Title/Abstract] OR ivf-et[Title/Abstract] OR IVF[Title/Abstract] OR IVF-ET[Title/Abstract] OR fertile[Title/Abstract] OR infertile[Title/Abstract] OR fertility[Title/Abstract] OR infertility[Title/Abstract] OR fertil*[Title/Abstract] OR infertil*)) AND (acrosin[MeSH] OR Acrosin*[Title/Abstract] OR acrosin enzyme[Title/Abstract] OR acrosin enzyme*[Title/Abstract] OR acrosomal enzyme[Title/Abstract] OR acrosomal enzyme*[Title/Abstract] OR acrosome enzyme[Title/Abstract] OR acrosome enzyme*[Title/Abstract] OR sperm acrosin[Title/Abstract] OR sperm acrosin*[Title/Abstract] OR human acrosin[Title/Abstract] OR human acrosin*[Title/Abstract] OR Akrosin*[Title/Abstract] OR Kennedy acrosin*[Title/Abstract] OR Acrosome proteinase[Title/Abstract] OR Acrosome proteinase*[Title/Abstract] OR Acrosomal proteinase[Title/Abstract] OR Acrosomal proteinase*[Title/Abstract] OR M beta-Acrosin[Title/Abstract] OR M beta-Acrosin* [Title/Abstract] OR M beta Acrosin[Title/Abstract] OR M beta Acrosin* [Title/Abstract] OR “proteinase, Acrosomal” [Title/Abstract] OR “beta-Acrosin, M” [Title/Abstract] OR “apical body enzyme” [Title/Abstract])) AND (detect*[Title/Abstract] OR assess*[Title/Abstract] OR evaluat*[Title/Abstract] OR assa*[Title/Abstract] OR test[Title/Abstract] OR determin*[Title/Abstract] OR predict*[Title/Abstract] OR predict* value*[Title/Abstract] OR predict* (nN) value*[Title/Abstract] OR predictive value of tests[MeSH] OR diagnos*[Title/Abstract] OR accurac*[Title/Abstract] OR diagnostic accurac*[Title/Abstract] OR sensitiv*[Title/Abstract] OR specificit*[Title/Abstract] OR sensitivity and specificity[MeSH] OR positive predictive value*[Title/Abstract] OR PPV*[Title/Abstract] OR negative predictive value*[Title/Abstract] OR NPV*[Title/Abstract] OR logistic*[Title/Abstract] OR regression*[Title/Abstract] OR spearman*[Title/Abstract] OR pearson*[Title/Abstract] OR r[Title/Abstract] OR correlat*[Title/Abstract] OR relat*[Title/Abstract] OR cut-off[Title/Abstract])

**AR assay**

The search strategy in Pubmed consisted of the following phrases:

(((Fertilization in Vitro[Mesh] OR Fertilization in Vitro[Title/Abstract] OR Fertilizations in Vitro[Title/Abstract] OR In Vitro Fertilization[Title/Abstract] OR In Vitro Fertilizations[Title/Abstract] OR in-Vitro Fertilization[Title/Abstract] OR in-Vitro Fertilizations[Title/Abstract] OR Test-Tube Fertilization[Title/Abstract] OR Test-Tube Fertilizations[Title/Abstract] OR Fertilization, Test-Tube[Title/Abstract] OR Fertilizations, Test-Tube[Title/Abstract] OR Test Tube Fertilization[Title/Abstract] OR Test Tube Fertilizations[Title/Abstract] OR Test Tube Baby[Title/Abstract] OR Test Tube Babies[Title/Abstract] OR Test-Tube Baby[Title/Abstract] OR Test-Tube Babies[Title/Abstract] OR Baby, Test-Tube[Title/Abstract] OR Babies, Test-Tube[Title/Abstract] OR Test tube baby technique[Title/Abstract] OR Test tube babies technique[Title/Abstract] OR Test tube ovum fertilization[Title/Abstract] OR Test tube ovum fertilizations[Title/Abstract] OR artificial fertilization[Title/Abstract] OR artificial fertilizations[Title/Abstract] OR ivf[Title/Abstract] OR ivf-et[Title/Abstract] OR fertile[Title/Abstract] OR infertile[Title/Abstract] OR fertility[Title/Abstract] OR infertility[Title/Abstract] OR fertil*[Title/Abstract] OR infertil*[Title/Abstract] OR fecund*[Title/Abstract] OR infecund*[Title/Abstract])) AND (acrosome reaction[Mesh] OR Exocytosis[Mesh] OR acrosome reaction[Title/Abstract] OR acrosome reaction*[Title/Abstract] OR exocytosis[Title/Abstract] OR acrosome status[Title/Abstract] OR acrosomal status[Title/Abstract] OR intact acrosome[Title/Abstract] OR acrosome intact*[Title/Abstract] OR acrosome integrity[Title/Abstract] OR acrosomal integrity[Title/Abstract] OR integrity of acrosome[Title/Abstract] OR reaction of acrosome[Title/Abstract] OR reaction* of acrosome[Title/Abstract] OR acrosome exocytosis[Title/Abstract] OR acrosomal exocytosis[Title/Abstract] OR spontaneous acrosome reaction*[Title/Abstract] OR stimulat* acrosome reaction* [Title/Abstract] OR acrosome reaction* stimulat*[Title/Abstract] OR induc* acrosome reaction*[Title/Abstract] OR acrosome reaction* induc*[Title/Abstract] OR acrosome reaction*, spontane*[Title/Abstract] OR acrosome reaction*, induc*[Title/Abstract] OR stimulat* acrosome reaction[Title/Abstract] OR acrosome reaction stimulat*[Title/Abstract])) AND (detect*[Title/Abstract] OR assess*[Title/Abstract] OR evaluat*[Title/Abstract] OR assa*[Title/Abstract] OR test* [Title/Abstract] OR determ*[Title/Abstract] OR examin*[Title/Abstract] OR measur* [Title/Abstract] OR monitor* [Title/Abstract] OR reliab*[Title/Abstract] OR predict*[Title/Abstract] OR predict* value*[Title/Abstract] OR predict* (nN) value*[Title/Abstract] OR predictive value of tests[Mesh] OR reference value*[Title/Abstract] OR diagnos*[Title/Abstract] OR accura*[Title/Abstract] OR diagnostic accura*[Title/Abstract] OR Diagnosis, Differential[Mesh] OR Diagnostic Errors[Mesh] OR scor*[Title/Abstract] OR sensitiv*[Title/Abstract] OR specific*[Title/Abstract] OR sensitivity and specificity[Mesh] OR Mass Screening[Mesh] OR Screen*[Title/Abstract] OR positive predictive value*[Title/Abstract] OR PPV*[Title/Abstract] OR positive*[Title/Abstract] OR False Positive Reactions[Mesh] OR negative predictive value*[Title/Abstract] OR NPV*[Title/Abstract] OR negative*[Title/Abstract] OR False Negative Reactions[Mesh] OR logistic*[Title/Abstract] OR regression*[Title/Abstract] OR Regression Analysis[Mesh] OR spearman*[Title/Abstract] OR pearson*[Title/Abstract] OR r[Title/Abstract] OR correlat*[Title/Abstract] OR relat*[Title/Abstract] OR Probability[Mesh] OR probabilit*[Title/Abstract] OR likelihood ratio*[Title/Abstract] OR ROC curve[Mesh] OR roc[Title/Abstract] OR sroc[Title/Abstract] OR receive operat*[Title/Abstract] OR cut-off[Title/Abstract] OR threshold*[Title/Abstract] OR analy*[Title/Abstract])
