# Supplementary material for: Human sperm acrosome function assays are predictive of fertilization rate in vitro: a retrospective cohort study and meta-analysis
Source: Reprod Biol Endocrinol. 2018 Aug 24;16:81. doi: 10.1186/s12958-018-0398-y (PMC6109296; doi:10.1186/s12958-018-0398-y)
Supplement: Supplementary file 2 — Table S1. Spearman correlation between fertilization rate and baseline characteristics, and AE result. (DOCX 15 kb) [file 12958_2018_398_MOESM2_ESM.docx]

**Table S1** Spearman correlation between fertilization rate and baseline characteristics, and AE result

| **Variables** | **Fertilization rate** | |
| --- | --- | --- |
|  | **R** | ***P*** |
| Female age (years) | 0.070 | 0.059 |
| Male age (years) | 0.052 | 0.159 |
| MII oocytes (n) | 0.054 | 0.146 |
| Abstinence days (n) | -0.010 | 0.778 |
| Semen volume (mL) | 0.016 | 0.659 |
| Concentration (× 10^6^/mL) | 0.035 | 0.349 |
| Motility (%) | 0.046 | 0.212 |
| Forward progression motility (%) | 0.119 | 0.001 |
| Percentage of normal morphology (%) | 0.069 | 0.062 |
| Infertility duration (years) | 0.054 | 0.141 |
| AE levels (μIU/10^6^ sperm) | 0.075 | 0.042 |

*AE* acrosomal enzyme, *MII* meta-phase II
