# Supplementary material for: Human sperm acrosome function assays are predictive of fertilization rate in vitro: a retrospective cohort study and meta-analysis
Source: Reprod Biol Endocrinol. 2018 Aug 24;16:81. doi: 10.1186/s12958-018-0398-y (PMC6109296; doi:10.1186/s12958-018-0398-y)
Supplement: Supplementary file 3 — Table S2. Grouping of patients according to AE result. (DOCX 14 kb) [file 12958_2018_398_MOESM3_ESM.docx]

**Table S2** Grouping of patients according to AE result

|  | **Fertilization outcome** | |
| --- | --- | --- |
|  | **TFF** | **Fertilization success** |
| Positive (AE levels < 25 μIU/10^6^ sperm)  Negative (AE levels ≥ 25 μIU/10^6^ sperm) | 30 | 587 |
|  | 4 | 116 |

*AE* acrosomal enzyme, *TFF* total fertilization failure
